# Synthetically controlling dendrimer flexibility improves delivery of large plasmid DNA†
†Electronic supplementary information (ESI) available: Detailed methods, chemical and cell-based characterization. See DOI: 10.1039/c7sc00097a



**DOI:** 10.1039/c7sc00097a

**Published:** 2017-01-27

**Authors:** Jessica A. Kretzmann, Diwei Ho, Cameron W. Evans, Janice H. C. Plani-Lam, Benjamin Garcia-Bloj, A. Elaaf Mohamed, Megan L. O'Mara, Ethan Ford, Dennis E. K. Tan, Ryan Lister, Pilar Blancafort, Marck Norret, K. Swaminathan Iyer

**Affiliations:** a School of Molecular Sciences , The University of Western Australia , 35 Stirling Hwy , Crawley , WA 6009 , Australia . Email: marck.norret@uwa.edu.au ; Email: swaminatha.iyer@uwa.edu.au; b Harry Perkins Institute of Medical Research , 6 Verdun St , Nedlands , WA 6009 , Australia . Email: pilar.blancafort@uwa.edu.au; c Research School of Chemistry , Australian National University , Canberra , ACT 2601 , Australia; d ARC Centre of Excellence in Plant Energy Biology , The University of Western Australia , 35 Stirling Hwy , Crawley , WA 6009 , Australia; e School of Anatomy, Physiology and Human Biology , The University of Western Australia , 35 Stirling Hwy , Crawley , WA 6009 , Australia

## Abstract

Tools for editing the genome and epigenome have revolutionised the field of molecular biology and represent a new frontier in targeted therapeutic intervention. Although efficiencies and specificities of genome editing technologies have improved with the development of TALEs and CRISPR platforms, intracellular delivery of these larger constructs still remains a challenge using existing delivery agents. Viral vectors, including lentiviruses and adeno-associated viruses, as well as some non-viral strategies, such as cationic polymers and liposomes, are limited by packaging capacity, poor delivery, toxicity, and immunogenicity. We report a highly controlled synthetic strategy to engineer a flexible dendritic polymer using click chemistry to overcome the aforementioned delivery challenges associated with genome engineering technologies. Using a systematic approach, we demonstrate that high transfection efficiencies and packaging capacity can be achieved using this non-viral delivery methodology to deliver zinc fingers, TALEs and CRISPR/dCas9 platforms.

## Abstract 2

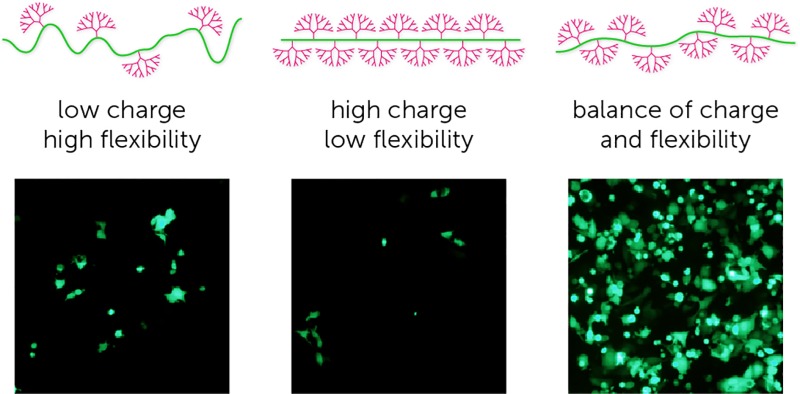
Tools for editing the genome and epigenome have revolutionised the field of molecular biology and represent a new frontier in targeted therapeutic intervention.

## Introduction

Emerging genome and epigenome technologies have the potential to correct mutations associated with disease, reactivate genes for therapy, and repress deleterious genes or sequences, but achieving efficient and reliable delivery of these tools remains a challenge.^1,2^ Modulation of gene expression can be achieved using three major classes of genomic editing tools: zinc finger proteins (ZFs), transcription activator-like effectors (TALEs), and the clustered regularly interspaced short palindromic repeat (CRISPR) associated protein (CRISPR/Cas9).^3^ The successful implementation of gene therapies will depend upon both high specificity for the target gene and high transfection efficiency to target cells. Zinc finger proteins can be easily packaged and delivered due to their small size, but have demonstrated broad off-target effects;^4^ TALEs can be programmed to almost any given DNA sequence with high specificity,^5^ but large plasmid size hinders successful delivery. Further development has produced the current state-of-the-art CRISPR system, which has potential for altering gene expression with high targeting density, ease of engineering for multiple targets, and minimal off-target effects.^6^ Effective CRISPR-based genome and epigenome editing is achieved *via* concurrent delivery of multiple components, including Cas9 or dCas9 plasmids and multiple short guide RNAs (sgRNAs), and in recent work this has necessitated the use of both liposomal and viral vectors to achieve cotransfection *in vivo*.^2,7^ Delivery systems based on lentiviral and adeno-associated viral vectors are limited by their intrinsic packaging capacity, whereas liposomes are limited by variability in forming DNA/liposome complexes, high toxicity, poor stability, and rapid clearance.^8,9^ Attempts to overcome these problems have involved using higher-capacity adenoviruses, smaller CRISPR constructs^10^ and hydrodynamic injection-based delivery^11^ strategies, albeit with lower editing efficiencies, a restricted range of accessible targets, and associated immunogenicity.^8,9^

Cationic macromolecules are promising gene delivery vectors due to their ability to condense plasmid DNA (pDNA) and protect it from cellular and restriction nucleases. Poly(ethylene imine) (PEI) is one of the most comprehensively studied polymeric materials for gene delivery, however transfection efficiency is poor for large plasmids, and correlates with substantial cytotoxicity.^9,12^ Comparisons of linear and randomly branched architectures to vary charge density and molecular weight have produced only modest improvements in transfection efficiency and toxicity.^13,14^ Among the various cationic macromolecules explored so far, the unique molecular structure of dendrimers offers high synthetic control to precisely regulate size and structure.^15^ Poly(amido amine) (PAMAM) dendrimers are the most widely used, commercially available, dendrimer-based non-viral vectors.^16^ The two important properties of a PAMAM dendrimer governing efficiency as a delivery agent are: (i) the high density of primary amines on the periphery, which interact with anionic DNA molecules to form stable polyplexes; and (ii) the high density of tertiary amines in the interior, which provide sufficient buffering capacity to enable endosomal escape of delivered DNA.^16^ However, with increasing generation, dendrimers become conformationally restricted and experience significant intermolecular interactions, which impairs their ability to form stable polyplexes with pDNA.^17,18^ Additionally, high-generation dendrimers are associated with significant cytotoxicity.^19,20^ These generation-dependent steric properties and toxicity impair the ability of native dendrimers to deliver large genome editing DNA constructs. The ideal dendrimer-based architecture for the delivery of large DNA constructs requires a high degree of flexibility and sufficient cationic charge to enable high DNA packing density for intracellular delivery, and sufficient tertiary amines to facilitate intracellular release of the cargo.^21^

Given that size, topology, and charge density are important factors in determining the transfection ability of a dendritic agent, we present a highly controlled synthetic strategy using click chemistry to engineer a library of dendritic copolymers.^19,21^ We demonstrate using this synthetic approach that it is possible to methodically tune and optimise the platform for transfection of both small and large plasmids. Importantly, we show that this design strategy overcomes the challenges associated with the traditional cationic macromolecules in the delivery of large genome editing tools with high efficiency.

## Results and discussion

### Synthesis of dendronised polymer and optimisation of transfection using small pDNA

**Fig. 1:**
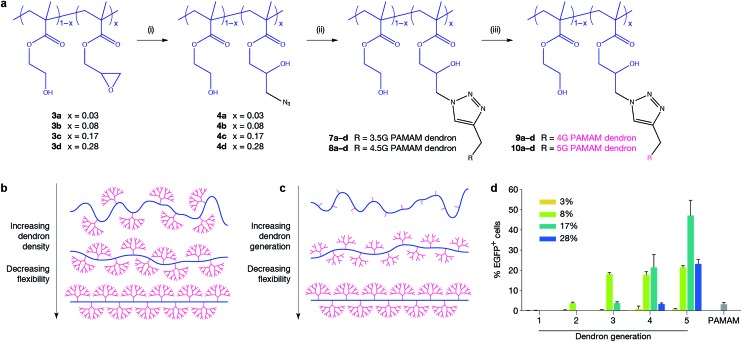
Polymer design and transfection efficiency in MCF-7 cells. (a) Synthesis of systematic dendronised polymer design and architecture; polymers are random statistical copolymers of HEMA and GMA where *x* = mol% GMA. Poly(amido amine) dendrons of different generation are clicked onto the linear polymer backbone. Reaction conditions: (i) NaN_3_, NH_4_Cl, DMF, 60 °C, 72 h; (ii) PMDETA, CuBr, DMF, r.t., 72 h; (iii) ethylenediamine, MeOH, 0 °C. (b and c) Representations of polymers prepared in this work. Schematics demonstrate how flexibility is systematically altered by varying dendron generation and dendron density independently. (d) Transfection efficiency of polymers delivering EGFP-encoding plasmid to MCF-7 cells at an N/P ratio of 10. Polymer **10c** (*x* = 0.17, G5 PAMAM dendron) significantly outperformed traditional G5 PAMAM dendrimer (*p* ≤ 0.0001).

In order to optimise the design of an efficient transfection agent, we adopted a synthetic strategy in which a linear copolymer backbone was used to anchor dendrons of varying generations (G1–G5) using copper-catalysed click chemistry. We expected that the ideal architecture would incorporate flexibility to allow for a high packing density, reduce the charge density to minimise cytotoxicity, and maintain the key chemical characteristics of higher-generation dendrimers. Given the widespread use of poly(2-hydroxyethyl methacrylate) in biomedical applications, in this work we chose 2-hydroxyethyl methacrylate (HEMA) as a spacer between glycidyl methacrylate (GMA) units to provide hydrophilicity, flexibility and biocompatibility.^22^ The GMA monomer is incorporated to enable easy azido functionalisation of the linear backbone and subsequent attachment of alkyne-functionalised dendrons using an azide/alkyne click reaction. The linear anchoring backbone was formed using atom-transfer radical-polymerisation (ATRP) of hydroxyethyl methacrylate (HEMA, **1**) and glycidyl methacrylate (GMA, **2**). PAMAM dendrons were selected for this study to fulfill the requirements of low cytotoxicity, controlled and quantifiable synthesis, and a known ratio of primary to tertiary amines. Changing the density of dendrons along the backbone (and hence flexibility of the system) and dendron generation were parameters to be investigated for their effect on transfection. Four different linear polymer backbones of poly[(2-hydroxyethyl methacrylate)-*ran*-(glycidyl methacrylate)] with varying GMA content (**3a**, *x* = 0.03; **3b**, *x* = 0.08; **3c**, *x* = 0.17; and **3d**, *x* = 0.28) were prepared and dendrons of various generations were attached to produce a library of dendronised polymers (**9a–10d**, Fig. 1a, ESI Table 1†). Our findings show that at high ratios of GMA, dendron attachment is limited by steric bulk (ESI Table 8†). Therefore, using a click reaction is advantageous as it allows for simple, controlled attachment of complete dendrons onto the backbone,^23,24^ enabling the synthesis of a library of well-defined dendronised polymers. Copolymer backbones were structurally characterised using NMR and GPC (ESI Table 1†). Gel retardation assays, DLS, and zeta potential measurements were used to determine optimal nitrogen-to-phosphorus (N/P) ratios for pDNA binding (ESI Fig. 6, 13, and 14† respectively). The calculation of N/P ratios were based on elemental analysis data (for nitrogen content, ESI Tables 6 and 7†) and calculated pDNA phosphorus content. Polymer formulations were screened at two different N/P ratios (ESI Fig. 7 and 8†). The ability of the polymers to transfect a small EGFP-encoding pDNA (5.3 kb) was first evaluated in the MCF-7 human breast adenocarcinoma cell line, which is regarded as difficult-to-transfect,^25^ against G5 PAMAM dendrimer as a control. The transfection efficiency was evaluated for all polyplexes at an optimised N/P ratio of 10 using fluorescent microscopy and quantified *via* flow cytometry (Fig. 1d). Our novel polymer architecture with G4 and G5 PAMAM dendrons demonstrated superior transfection compared to the traditional G5 PAMAM dendrimer. G1, G2 and G3 dendronised polymers achieved transfection levels similar to native dendrimers (<20%) and were not pursued further. The study revealed strong relationships between transfection efficiency and dendron generation (G5 dendronised polymers **10a–d** showed the highest transfection efficiencies) and between transfection efficiency and dendron density on the backbone (**10c** outperformed **10a**, **10b**, and **10d**). To explain the observed drop in transfection efficiency between polymers **9c** and **9d**, and between **10c** and **10d**, we hypothesise that steric repulsion of highly substituted backbones limits the flexibility of the backbone (Fig. 1b), potentially interfering with electrostatic pDNA complexation. Molecular dynamics simulations of a linear HEMA backbone substituted with two G1 PAMAM dendrons, a small-scale representation of our polymer system, supports this argument (ESI Fig. 10–12†). When the distance between the dendrons is short, corresponding to a high degree of substitution, steric repulsion between dendrons limits the conformational flexibility of the system.

### Effect of fluorination: delivery of large pDNA

**Fig. 2:**
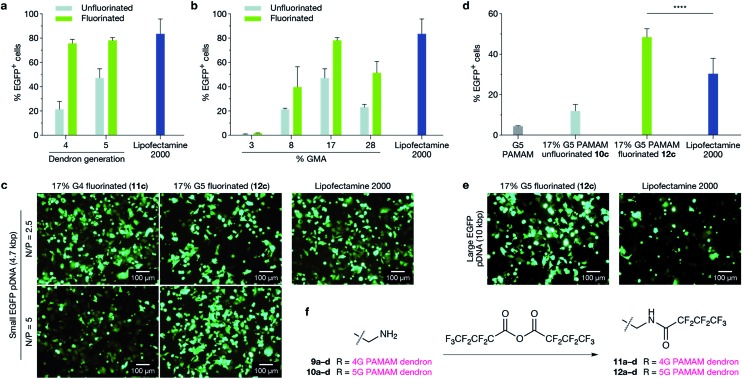
Transfection efficiency in MCF-7 cells comparing the effect of perfluoroalkyl modification of polymers on both small and large plasmids. (a and b) Fluorination of dendronised polymers increased EGFP-encoding pDNA transfection efficiency significantly in all cases ((a) *p* ≤ 0.0001; (b) *p* ≤ 0.001) and fluorinated dendronised polymers **11c** and **12c** achieved transfection efficiencies similar to Lipofectamine 2000 (*p* > 0.05). In (a), G4 and G5 PAMAM dendrons (**9c**, **10c**, **11c** and **12c**) are compared, and in (b), G5 PAMAM dendronised polymers (**10a–d** and **12a–d**) are compared. (c) Representative fluorescence microscopy images of transfection efficiencies in MCF-7 cells with fluorinated dendronised polymers **11c** and **12c** at N/P = 2.5 and 5 compared to Lipofectamine 2000. (d) MCF-7 cells were transfected with a large EGFP-expressing plasmid (10.3 kb); transfection efficiency was quantified with flow cytometry. Polymer **12c** achieved a significantly higher transfection efficiency than Lipofectamine 2000 (*p* ≤ 0.0001). (e) Delivery of large EGFP plasmid to MCF-7 cells using fluorinated dendronised polymer **12c** compared to Lipofectamine 2000. (f) Reaction scheme for fluorination of terminal PAMAM primary amines using heptafluorobutyric anhydride. Dendronised polymers **9a–10d** were all treated to give fluorinated dendronised polymers **11a–12d** using the same method.

Having established that G5-dendron-substituted polymers **10b–d** performed better than conventional G5 PAMAM dendrimers and resulted in no detectable cytotoxicity (ESI Fig. 9†), we investigated the effect of fluorination on the transfection efficiency of G5-dendron substituted polymers. It has been demonstrated that fluorination enhances cellular uptake of polyplexes, facilitates their endosomal escape and provides excellent serum resistance.^26^ The extent of fluorination was kept consistent to previously reported numbers that demonstrated no cytotoxicity.^26^ No detectable toxicity was observed in our polymers following fluorination (ESI Fig. 19†). We next compared the ability of unfluorinated **10a–d** and fluorinated **12a–d** G5 dendronised polymers to transfect EGFP-encoding pDNA (5.3 kb) in MCF-7 cells against Lipofectamine 2000 as a control (Fig. 2a). Optimal N/P ratios were again determined by gel retardation assays, DLS, and zeta potential measurements (ESI Fig. 13 and 14†) and screening at three different N/P ratios (ESI Fig. 15–18†). Lipofectamine 2000 was used in this evaluation as a positive control as it is a widely used, commercially-available reagent with consistently high transfection efficiencies. Fluorination enhanced the transfection ability of G5 dendronised polymers for all polymer backbones, and polymer **12c** displayed transfection efficiencies similar to Lipofectamine 2000 (Fig. 2b). To further demonstrate that our novel dendritic polymer architecture can perform effective packaging and delivery of large expression cassettes, we next evaluated delivery of a larger EGFP-encoding pDNA (10.3 kb). The fluorinated dendronised polymer **12c** outperformed both G5 PAMAM dendrimers and Lipofectamine 2000 for delivery of large pDNA (Fig. 2d and e).

### Delivery of multiple plasmids

**Fig. 3:**
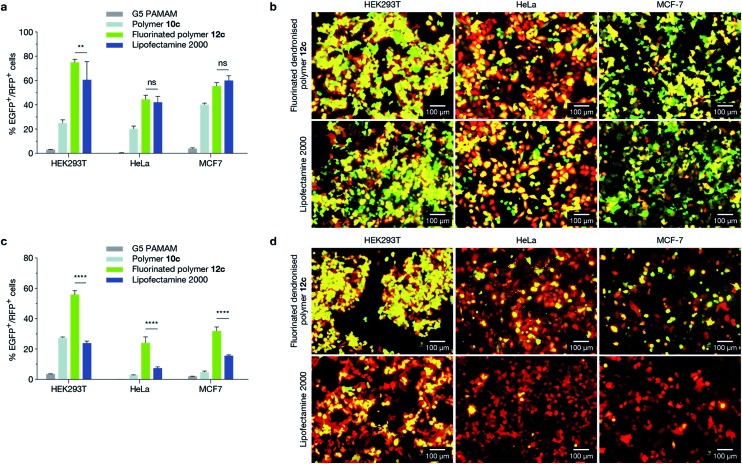
Cotransfection experiments in HEK293T, HeLa and MCF-7 cell lines. (a and b) Cell lines were cotransfected with two pDNAs of equal size encoding EGFP and mCherry (both 5.3 kb). (c and d) Cell lines were cotransfected with plasmids of different size encoding EGFP (10.3 kb) and mCherry (5.3 kb). Cotransfection efficiency was measured using flow cytometry (a and c), and visualised using epifluorescence microscopy (b and d) where concurrent expression of both plasmids appears yellow. The fluorinated dendronised polymer **12c** formulation demonstrated significantly higher cotransfection levels of multiple-sized plasmids compared to Lipofectamine 2000 across all tested cell lines (*p* ≤ 0.0001).

We next evaluated the ability of **12c** (17%, 5G fluorinated polymer) to perform cotransfections in HEK293T (human embryonic kidney), HeLa (human cervical carcinoma), and MCF-7 cell lines with two distinct pDNAs of similar size (Fig. 3a and b), encoding EGFP (5.3 kb) and mCherry (5.3 kb), as well as with pDNAs of different size (Fig. 3c and d), encoding EGFP (10.3 kb) and mCherry (5.3 kb). HEK293T and HeLa cell lines were chosen as they are widely used model cell lines, and results were compared to a traditional G5 PAMAM dendrimer. We confirmed the absence of cytotoxicity of the polymer in HEK293T and HeLa cell lines (ESI Fig. 27†). It is evident that **12c** outperformed both Lipofectamine 2000 and G5 PAMAM dendrimer for the delivery of mixed pDNA in all tested cell lines (Fig. 3). This further consolidated the theory that while conventional liposomal transfection agents are capable of delivering pDNA of lower sizes, they are limited in their packaging capacity for delivering large expression cassettes. The novel design strategy proposed in the current study can overcome this limitation.

The literature generally shows that among PAMAM dendrimers, G4–7 are most effective for delivery of pDNA.^27^ This is consistent with our observations, and is likely attributable to an optimal balance between primary amine charge density and tertiary amine buffering ability. Our data shows that polymer **12c** results in the best transfection efficiency. We suggest that: (i) there is an optimal dendron density and level of flexibility shown by the **10c** polymer formulation that maximises the interaction between dendrons and (multiple) pDNAs, forming stable polyplexes; (ii) there is a balance between charge density and buffering ability for plasmid binding and release, which is given by the 5G dendron; and (iii) fluorination facilitates the internalisation and release of polyplexes. While previous studies have used G4–7 PAMAM dendrimers to try to balance charge density and buffering ability,^27^ the restrictions imposed by spherical architectures (including branch flexibility) have not previously allowed each of these factors to be varied independently.

### Delivery of genome editing tools

**Fig. 4:**
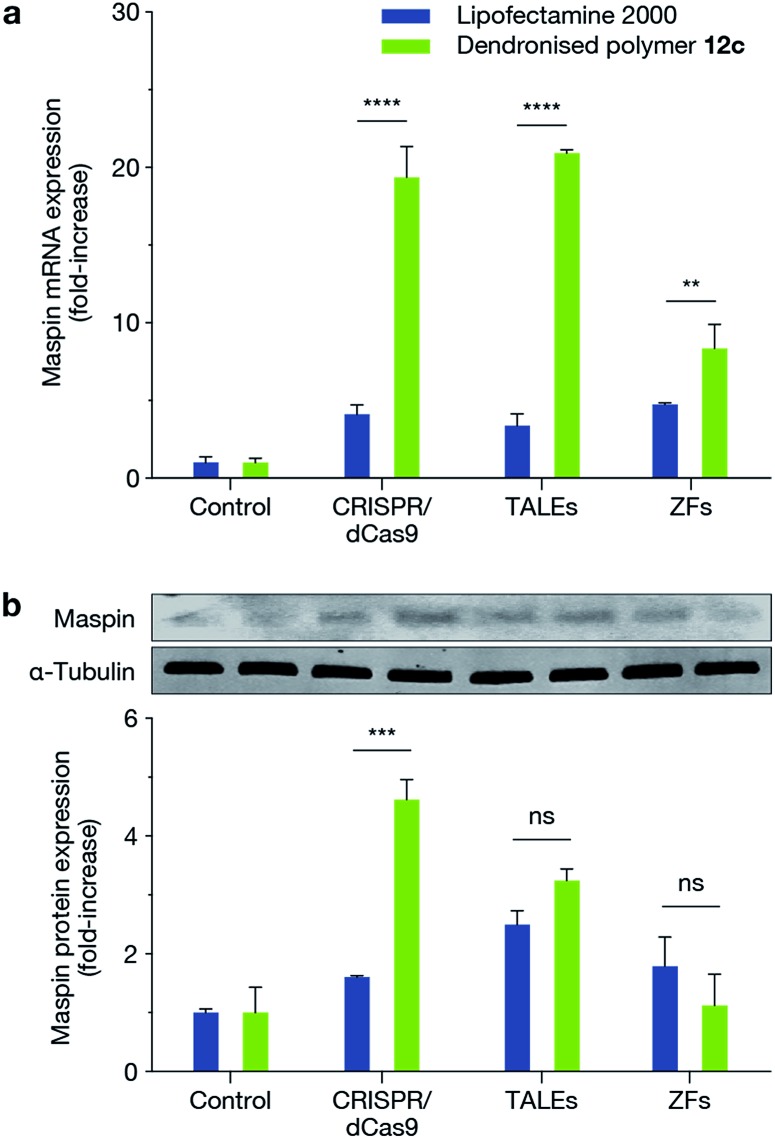
Transfection of MCF-7 cells with CRISPR/dCas9, TALEs and ZFs using VP64 effector for the activation of MASPIN. Transfection using polymer **12c** resulted in increased MASPIN mRNA (a) across all platforms and increased MASPIN protein (b) levels for CRISPR/dCas9 compared to Lipofectamine 2000 (**p ≤ 0.01, ***p ≤ 0.001, ****p ≤ 0.0001, ns *p* > 0.05). Cells were transfected with an empty plasmid as a control.

We next demonstrated the ability of this novel dendritic architecture to achieve a functional outcome by delivering three different genome engineering tools: CRISPR/dCas9-VP64 with a pool of sgRNAs (dCas9-VP64: 9.8 kb; sgRNAs: 3.2 kb), TALE-VP64 (9.3 kb) and ZF-VP64 (6.1 kb) for transcriptional activation of MASPIN (mammary serine protease inhibitor) in the MCF-7 cell line. MASPIN is a tumour suppressor gene and was chosen as a model target for the present study, though its role as a central regulator of tumour progression is still debatable.^28,29^ Loss of MASPIN expression is associated with increased invasive potential and metastasis.^29,30^ Delivery of each of the above genome engineering tools for transcriptional activation of MASPIN was achieved using the fluorinated dendronised polymer **12c** and compared against Lipofectamine 2000 as control. Significant activation was achieved for all platforms at the mRNA level and for CRISPR/dCas9-VP64 at the protein level (*p* ≤ 0.001) (Fig. 4).

The evolution of genome editing tools in the recent past has resulted in technologies such as the CRISPR system, which is capable of concurrent targeting of distinct genomic loci, but requires simultaneous transfection of multiple plasmids. The cotransfection of multiple plasmids is a major challenge, particularly when transfection efficiency is poor, leading to highly variable expression levels. Our data shows that Lipofectamine 2000 can deliver small (∼5 kb) pDNA well, but fails to deliver large (∼10.3 kb) pDNA with similar efficiency. We speculate that the liposomal curvature is constrained by the positively charged head group, restricting liposome size and preventing efficient encapsulation of large plasmids. This argument is supported by previous data showing that the physicochemical properties of lipoplexes are dependent only on the chemical composition of the liposomes and not on pDNA size.^31^ Using our dendronised polymers, however, we achieved successful transfection of large pDNA. We observed that co-expression of plasmids in difficult-to-transfect cells was higher when plasmids were mixed and co-delivered, in comparison to complexing and delivering the plasmids separately (ESI Fig. 23†). Plasmids can be successfully delivered separately only when high transfection efficiency (∼100%) is achievable, as demonstrated recently in HEK293T cells and liver cells *in vivo*.^7^ However, for less ideal systems in which the transfection efficiency is lower, we find that it is beneficial to mix all components of gene editing systems with a single vector. This requires a system that can bind both large and small plasmids.

This same reasoning is reflected in the results of our functional study where we demonstrated the delivery of ZF-VP64, TALE-VP64 and CRISPR/dCas9-VP64. Among these three epigenome editing technologies, the sequence encoding the ZF proteins is relatively short and is therefore not likely to be limited by vector capacities. However, ZFs suffer from off-target effects due to a short recognition motif, strong preference for CG-rich sequences, and are limited in the number of sites that they can effectively and selectively target. These shortcomings are overcome by the more flexible TALE and CRISPR technologies, but the increased size of these tools and the requirement for concurrent delivery of multiple components make their introduction into cells a greater challenge, and is currently a bottleneck in the development of gene therapies. We used large plasmids and multiple plasmids encoding fluorescent proteins to model and address this issue, and demonstrated significantly improved efficiencies using our fluorinated dendronised polymer **12c**, as compared to Lipofectamine 2000. Delivery of ZF-VP64, TALE-VP64 and CRISPR/dCas9-VP64 designed to upregulate the tumour suppressor MASPIN further validated these findings. Using our optimised polymer design, we achieved significant upregulation of MASPIN at the mRNA and protein levels over the widely used Lipofectamine 2000.

## Conclusions

We have shown that linear dendronised polymers represent a controllable, synthetic platform that has allowed us to systematically engineer a highly efficient and nontoxic agent for the delivery of precise gene editing tools such as CRISPR and TALEs. Although rapid advances are being made both in designing novel genome-editing tools with increased specificity whilst limiting off-target effects, the development of delivery technologies that cater for large plasmid sizes associated with these technologies has been limited. This limitation has to be overcome for successful clinical translation of these technologies. Herein, we have shown that by carefully redesigning the architecture of dendrimers, it is possible to overcome both the packaging capacity and cytotoxicity issues associated with higher generation dendrimers, whilst maintaining the key attractive features of traditional dendrimers, such as highly controlled synthesis. The delivery platform proposed in the current study offers the possibility of incorporating features of nanoscale therapeutic agents such as multimodality and targeted delivery for *in vivo* translation of the genome editing tools.

## Experimental

### General

Reactions were carried out under standard Schlenk conditions. Where specified, solutions were degassed by standard ‘freeze–pump–thaw’ cycles. 2-(Morpholino)ethyl 2-bromoisobutyrate (ME-Br) was prepared according to the method of Weaver *et al.*;^32^ all other chemicals were purchased from Sigma Aldrich.

### Backbone synthesis

Statistical poly(HEMA-*ran*-GMA) were synthesised by ATRP of **1** and **2**, adapted from reported methods.^32^ In a typical reaction, monomer inhibitors were removed using a basic alumina column. Monomers were dissolved in MeOH and degassed. Copper(i) bromide (0.70 mmol) was combined with 2,2′-bipyridine (2.5 mmol) and monomer solutions at various feed ratios (as detailed in ESI Table 1,† total monomer ∼32 mmol). ME-Br (1 mmol) initiator was added and reaction was carried out at 80 °C for 2 h. The product **3** was collected by repeated precipitation in diethyl ether and centrifuged; the solid product was dried overnight under vacuum.

### Dendron synthesis and attachment

Propargyl-poly(amido amine) dendron synthesis was adapted from published procedures.^33^ Azide functionalisation of poly(HEMA-*ran*-GMA) polymers was achieved by treating copolymer **3a** with sodium azide and ammonium chloride in DMF. The reaction was allowed to proceed at 60 °C for 72 h. The solution was cooled, insoluble byproducts were removed by centrifugation, and the product **4a** was purified by repetitive precipitation in ether and dried under vacuum. Other copolymers were treated the same way and reactions scaled accordingly. PAMAM dendrons were attached to the azido-functionalised polymers by a copper-catalysed alkyne–azide click reaction, adapted from Zhao *et al.* ^34^ For reaction with **4a**, 3.5G propargyl-PAMAM dendron (**5**) was dissolved in DMF before addition of **4a**. Pentamethyldiethylenetriamine was added and the solution was degassed. The reaction commenced at room temperature with the addition of copper(i) bromide and proceeded for 72 h. Product **7a** was purified by dialysis against deionised water and collected *via* lyophilisation. Dendron generation was completed by reaction with ethylenediamine, before being purified by dialysis against deionised water and collected by lyophilisation to yield product **9a**.

### Dendron fluorination

Fluorination of PAMAM dendrons was adapted from Wang *et al.* ^26^

### General transfection protocol

Polymer stock solutions were made to a concentration of 10 mM primary amines in sterile Milli-Q water. Cells were seeded in standard 12-well plates 16–24 h prior to transfection, at concentrations resulting in 50–60% confluency at time of transfection. Polymer solution and pDNA were diluted to working concentrations in Opti-MEM reduced serum media (Gibco). For cotransfection experiments, pDNAs were mixed at 1 : 1 mass ratio. Polymer and pDNA solutions were thoroughly mixed to achieve desired N/P ratio for 1 μg DNA, and incubated at r.t. for 30 min. Cells were washed with PBS to remove serum, and media was replaced with 300 μL Opti-MEM (Gibco). Transfection cocktails were added and cells were incubated for 4 h before addition of 570 μL complete culture medium. Cells were incubated for a further 44 h. Commercial transfection agent Lipofectamine® 2000 (Thermo Fisher Scientific), was used according to the manufacturer's protocol at an optimised ratio of 1 μL μg^–1^ pDNA. Transfection efficiency was assessed using EGFP and mCherry reporter vectors, visualised with epifluorescence microscopy (Olympus IX-51, U-MGFPHQ and U-MRFPHQ filters) and quantified with flow cytometry (BD FACSCanto II for EGFP experiments, BD LSRFortessa for cotransfections).

### Polyplex characterisation

Polymer stock solutions were made to a concentration of 10 mM primary amines in sterilised Milli-Q water. For gel retardation assays, polymer solutions were mixed with pDNA (50 ng) at various N/P ratios and incubated at r.t. for 30 min. Samples were electrophoresed on 1% w/v agarose gels in sodium borate buffer (pH 9) and stained with ethidium bromide. For DLS and zeta potential measurements (Zetasizer Nano ZS, Malvern, UK), polymer solutions were incubated with pDNA (1 μg) at room temperature for 30 min. Solutions were diluted to 1 mL and measurements were taken in triplicate after equilibration for 2 min. The intensity-weighted zeta potential and hydrodynamic radius of polyplexes were measured; all zeta potential measurements were taken at pH ≈ 6.

### Cytotoxicity

Cells were seeded and transfected in poly(l-lysine)-coated 96-well plates under conditions and at densities proportional to those above (‘General transfection protocol’), being adjusted for well growth area. After 48 h transfection, CellTiter-Glo® 2.0 (Promega) assay was used to quantify cell viability, in accordance with the manufacturer's instructions, except that only 10 μL reagent was added to each well. This was confirmed to still give a linear response with cell number (data not shown). Luminescence was recorded (PerkinElmer EnSpire) using 0.05 s measurement time averaged over 12 points per well and normalised to untreated controls.

## Author contributions

KSI, MN and JAK designed experiments and developed the concept. JAK and MN synthesised and characterised dendritic polymers. JAK, DH, CWE and JHCP-L performed *in vitro* transfection validation. BGB, DEKT, EF, RL, and PB developed the plasmids, designed the functional assays and mixed plasmid delivery experiments. AEM, JAK, and MLO performed the computational analysis. All the authors discussed the results, assisted with analysis and contributed to writing the manuscript.
