# Supplementary material for: Synthetically controlling dendrimer flexibility improves delivery of large plasmid DNA
Source: Chem Sci. 2017 Jan 27;8(4):2923–30. doi: 10.1039/c7sc00097a (PMC5376716; doi:10.1039/c7sc00097a)
Supplement: Supplementary file 1 [file SC-008-C7SC00097A-s001.pdf]

Electronic Supplementary Material (ESI) for Chemical Science.
This journal is © The Royal Society of Chemistry 2017

Supplementary Information

Synthetically controlling dendrimer flexibility improves delivery of large plasmid DNA

Jessica A. Kretzmann, Diwei Ho, Cameron W. Evans, Janice H.C. Plani-Lam,
Benjamín García-Bloj, A. Elaaf Mohamed, Megan L. O'Mara, Ethan Ford, Dennis E.K. Tan,
Ryan Lister, Pilar Blancafort*, Marck Norret* & K. Swaminathan Iyer*

* pilar.blancafort@uwa.edu.au, marck.norret@uwa.edu.au, swaminatha.iyer@uwa.edu.au

1 Methods 3
1.1 Chemical synthesis and characterisation 3
1.1.1 Instrumental characterisation methods 3
1.1.2 ATRP of copolymer 3
1.1.3 Click chemistry 4
1.1.4 Fluorination 5
1.2 Molecular dynamics 6
1.3 Plasmid Binding 6
1.3.1 Plasmids 6
1.3.2 Gel retardation assays 7
1.3.3 DLS and zeta potential optimisations 7
1.4 Cell culture and gene transfection 7
1.5 Cytotoxicity 8
1.6 Functional outcome transfection study 9
1.6.1 mRNA extraction and reverse-transcription PCR 9
1.6.2 Immunoblotting 9
1.7 Statistical Analysis 10

2 Supplemental Data 11
2.1 Polymer backbone characterisation 11
2.1.1 ^{1}H NMR spectra of polymer backbones 11
2.1.2 Monomer reactivity ratios 12
2.2 Dendronised polymer characterisation 13
2.2.1 Generation 4 dendronised polymer 13
2.2.2 Generation 5 dendronised polymer 15
2.2.3 IR spectra 17
2.2.4 Substitution Pattern 18
2.2.5 Molecular Weight 18
2.3 Optimisation of transfection: systematic analysis of backbone and dendron generation 18
2.3.1 Cytotoxicity 20
2.4 Molecular dynamics analysis 21
2.5 Fluorination of dendronised polymers 23
2.5.1 Elemental analysis 23
2.5.2 Fluorination percentage and molecular weights 24
2.6 Optimisation of transfection for small (5.3 kb) plasmid 25

2.6.1 Gel retardation assay 25
2.6.2 DLS/zeta optimisation 25
2.6.3 Representative transfection images (MCF-7) 26
2.6.4 Cytotoxicity 28
2.7 Optimisation of transfection for large (10.3 kb) plasmid 28
2.7.1 Gel retardation assay 28
2.7.2 DLS/Zeta measurements 29
2.7.3 Representative transfection images 30
2.8 Cotransfection 31
2.8.1 Method of codelivery 31
2.8.2 Cotransfection using plasmids of equal size 31
2.8.3 Cotransfection using plasmids of different size 32
2.8.4 Cotransfection flow cytometry gatings 32
2.8.5 Cytotoxicity in cotransfected cell lines 33
2.9 *MASPIN* mRNA quantification 33
2.10 *MASPIN* protein quantification 34
2.11 Plasmid maps 35

3 Summary of Prepared Compounds 37

1 Methods

1.1 Chemical synthesis and characterisation

All chemicals were purchased from Sigma Aldrich and used without further purification. Poly(amido amine) G5.0 dendrimer with an ethylene diamine core was purchased (cat. 536709) as a 5 wt% solution in methanol and was dialysed against distilled water and lyophilised prior to being used *in vitro*.

1.1.1 Instrumental characterisation methods

^{1}H and ^{19}F NMR spectra were measured using a Varian 400 MHz spectrometer and Bruker 500 MHz spectrometer, using CD_3OD as the solvent for copolymers, aminated copolymers, dendronised polymers and fluorinated compounds. The chemical shifts were referenced to the solvent peak, δ = 3.31 ppm for CD_3OD. For ^{19}F spectra, hexafluorobenzene was used as a standard, with a chemical shift of δ = -164.9 ppm. IR spectra were obtained using PerkinElmer Spectrum One FT-IR spectrometer. Gel permeation chromatography (GPC) was used to determine the molecular weight and polydispersity index of polymers (Waters Styragel HR 4 DMF 4.6 × 300 mm column, 5 µm). Agilent Technologies 1100 Series GPC and Agilent GPC software were used for measurements and data analysis respectively. Measurements were taken using DMF as the eluent at a flow rate of 0.3 mL/min at 50 °C, and calibrated against poly(methyl methacrylate) (PMMA) standard. Elemental analysis was conducted at the Campbell Microanalytical Laboratory, University of Otago. The carbon, hydrogen and nitrogen content of each sample were determined via the 'flash combustion' method using a Carlo Erba Elemental Analyser EA1108. The fluorine content of fluorinated polymers was determined via the standard addition method using a fluoride selective ion electrode connected to a Eutech 2700 pH meter.

1.1.2 ATRP of copolymer

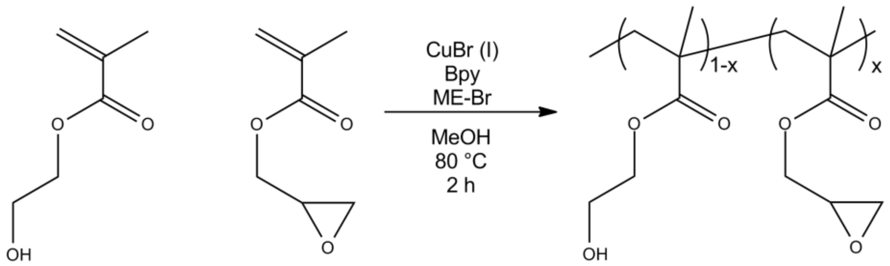

1 2 3

For a typical reaction, inhibitors for hydroxyethyl methacrylate (HEMA, **1**) and glycidyl methacrylate (GMA, **2**) were removed using a basic alumina column. Each monomer was dissolved in methanol (MeOH) at a ratio of 1:3 (monomer : MeOH) respectively. Prior to use, each monomer solution was degassed using standard 'freeze-pump-thaw' method and backfilled with nitrogen gas. Copper (I) bromide (CuBr, 100 mg, 0.70 mmol) was added to the flask, followed by 2,2'-bipyridine (bpy, 392 mg, 2.5 mmol) before monomer solutions were added at various ratios (Supplementary Table 1). 2-(4-Morpholino)ethyl 2-bromoisobutyrate initiator (ME-Br, 210 µL, 1 mmol) was added, and reaction was heated to 80 °C under standard Schlenk conditions for 2 h before reaction was opened to air and an additional MeOH (15 mL) added. The product was collected under reduced pressure and redissolved in minimal MeOH, then

collected by repeated precipitation in excess diethyl ether and centrifugation. Solid product was dried overnight under vacuum. Poly($HEMA_{1-x}$-*ran*-GMA_x) polymers (**3**) were obtained at ≈55% yield. Copolymer composition was determined by ^{1}H NMR (500 MHz, CD_3OD), where the appearance of peaks δH 2.70 (1H, br) and 2.87 (1H, br) correspond to the epoxide moiety, confirming presence of GMA. Molecular weight and PDI of polymers were measured using GPC (Supplementary Table 2) (Adapted from M. Smallridge *et al*.[1]).

Supplementary Table 1: Monomer feed ratios for ATRP reactions to produce poly(HEMA-*ran*-GMA) copolymers.

**Polymer**	**Feed ratios**		**GMA mol% (^{1}H integration)**
	**GMA/MeOH**	**HEMA/MeOH**	
**3a**	0.8 mL (1.5 mmol)	15.2 mL (31.2 mmol)	3.7
**3b**	1.9 mL (3.6 mmol)	14.1 mL (29.0 mmol)	8.9
**3c**	4 mL (7.5 mmol)	12 mL (24.7 mmol)	16.3
**3d**	6.4 mL (12 mmol)	9.6 mL (19.7 mmol)	27.9

Supplementary Table 2: Copolymer molecular weight and PDI measurements from GPC.

**Polymer**	**Mw (kDa)**	**PDI**
**3a**	13.6	1.25
**3b**	15.7	1.21
**3c**	21.5	1.30
**3d**	17.5	1.30

1.1.3 Click chemistry

Azido functionalisation: Copolymer **3a** (1.5 g, 0.34 mmol epoxide) was dissolved in dimethylformamide (DMF, 30 mL). Sodium azide (45 eq) and ammonium chloride (41 eq) were added to the stirring solution. Amounts were scaled accordingly for other polymer species. Reaction occurred at 60 °C for 72 h. Reaction solution was cooled and centrifuged to remove solid. Polymer was precipitated repeatedly in ether and dried under vacuum.

Click Reaction: For reaction with **3a**: 3.5 generation propargyl dendron (320 mg, 0.11 mmol, 2.4 eq) was dissolved in DMF (15 mL) before the addition of azido-functionalised copolymer **4a** (200 mg, 45.3 µmol epoxide). Both species were dissolved before the addition of pentamethyldiethylene triamine (PMDETA, 22.4 µL, 0.11 mmol). Reaction solution was degassed via 'freeze-pump-thaw' methods and backfilled with argon, before the reaction commenced under argon with the addition of CuBr (15.2 mg, 0.11 mmol). Reaction proceeded over 72 h at RT before being dialysed against DI water (4 × 4 L) and product **7a** collected via lyophilisation. **7a** was dissolved in minimal MeOH and added dropwise to a solution of excess ethylene diamine at 0 °C; reaction was left to proceed at room temperature for 7 d, then diluted with water and dialysed against DI water (4 x 4L) before being lyophilised to afford **9a** as a white solid. Amounts were scaled accordingly for other polymer and dendron species. (Method adapted from Zhao *et al*[2]).

[1] Weaver JVM, Bannister I, Robinson KL, Borles-Azeau X, Armes SP. Stimulus-responsive water-soluble polymers based on 2-hydroxylethyl methacrylate. *Macromolecules* 2004, 37(7), 2395–2403.

[2] Zhao P, Yan Y, Feng X, Liu L, Wang C, Chen Y. Highly efficient synthesis of polymer brushes with PEO and PCL as side chains via click chemistry. *Polymer* 2012, 53(10), 1992–2000.

Propargyl Dendron synthesis: Synthesis for PAMAM dendrons was adapted from methods presented by Lee *et al.* and Lin *et al.* [3]

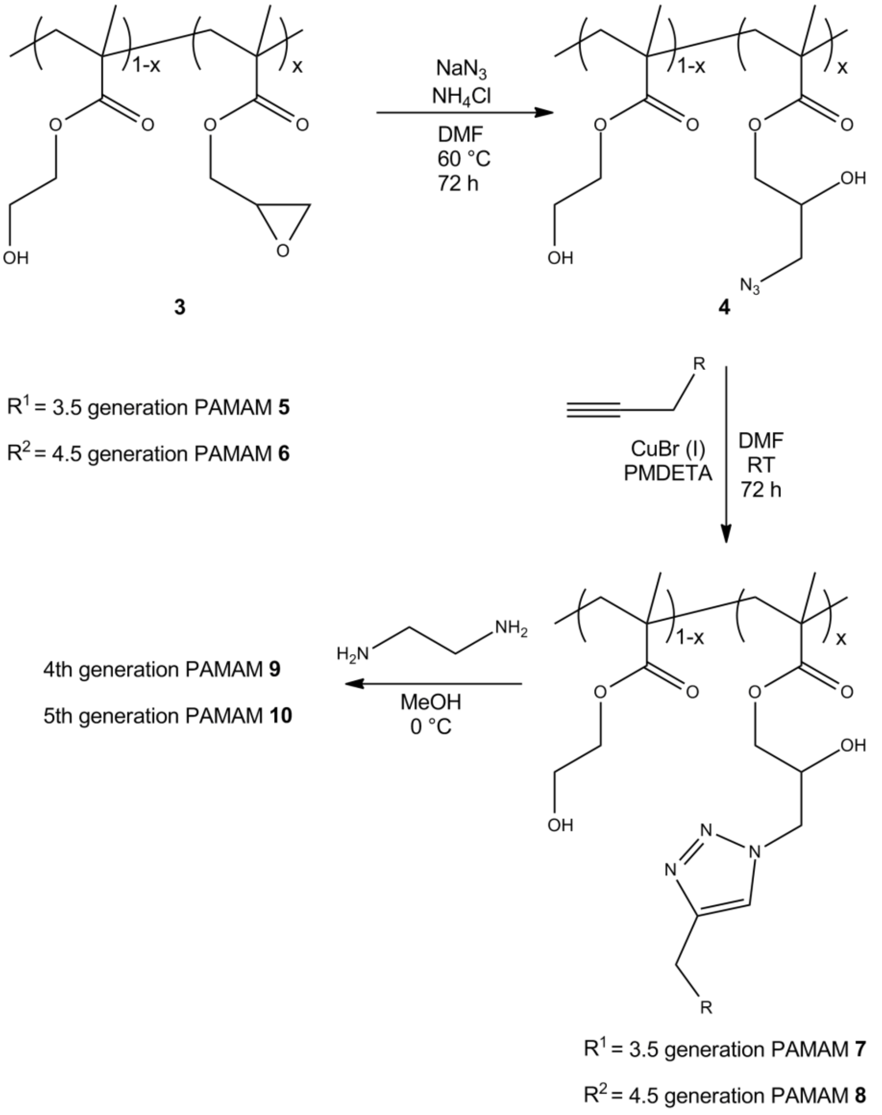

1.1.4 Fluorination

Polymers were fluorinated as per protocol described by Wang *et al.*[4]

---

[3] Lee JW, Kim B-K, Kim HJ, Han SC, Shin WS, Jin S-H. Convergent Synthesis of Symmetrical and Unsymmetrical PAMAM Dendrimers. *Macromolecules* 2006, 39(6), 2418–2422; Lin Y-J, Tsai B-K, Tu C-J, Jeng J, Chu C-C. Synthesis of β-cyclodextrin and poly(amido amine) dendron click cluster and its synergistic binding property. *Tetrahedron* 2013, 69(7), 1801–1807.

[4] Wang M, Liu H, Cheng Y. A fluorinated dendrimer achieves excellent gene transfection efficacy at extremely low nitrogen to phosphorus ratios. *Nat Commun* 2014, 5, 3053.

1.2 Molecular dynamics

All MD simulations were performed using GROMACS (Groningen Machine for Chemical Simulations) package,[5] version 4.6.5 with the GROMOS 54a7 forcefield.[6] All simulations were performed under periodic boundary conditions in a square box, with solvent (water) described using the simple point charge (SPC) water model.[7] For each system, the box size was chosen so that the minimum distance between any atom of the polymer and the box wall was at least 1.0 nm. Structures for each of the polymer fragments were drawn and converted to PDB (protein database) files using ChemDraw. Parameters for 2-hydroxyethyl methacrylate (HEMA) and glycidyl methacrylate (GMA) based on the GROMOS 54a7 forcefield were developed using the Automated Topology Builder (ATB).[8] Counterions (Cl^-) were included in each system ensure the overall charge neutrality. The LINCS algorithm[9] was used to constrain the lengths of the covalent bonds in the polymer. The geometry of the water molecules was constrained using the SETTLE algorithm.[10] Three model polymer systems were developed for use in MD simulations. The Control System contained a 10 HEMA polymer, $(HEMA)_{10}$. Systems 1 and 2 were also 10 fragment polymers: System 1 contained (HEMA)-(GMA)-$(HEMA)_6$-(GMA)-(HEMA) and System 2 contained $(HEMA)_3$-(GMA)-$(HEMA)_3$-(GMA)-$(HEMA)_3$. Each system was energy minimised for 1000 steps using a steepest descent algorithm. Simulations were conducted in the NPT-ensemble at T = 300 K and P = 1 bar; pressure coupling was isotropic. Three simulations were run for 100 ns, giving a total of 300 ns simulation time for each system, with data collected every 50 ps. Images were produced using VMD software, graphs using Xmgrace.

1.3 Plasmid Binding

1.3.1 Plasmids

The pcDNA3.1-dCas9-VP64 (Cat. No.: 47107, Addgene) plasmid, with a CMV promoter and VP64 at the C-terminal, was provided by Charles Gersbach (Duke University, Durham, USA).[1] The pcDNA-dCas9-No Effector with HA tagged at the C-terminus was purchased from Addgene (Cat. No: 47106, Addgene). All dCas9 vectors co-transfected with four single guided RNAs (sgRNAs) with custom-designed modification against MASPIN target sequences in *MASPIN* proximal promoters, using the crispr.mit.edu website tool and cloned into BbsI sites in the pSP-gRNA (sgRNA) expression vector (Cat No: 47108, Addgene), following Gersbach's protocol.[11] All dCas9

---

[5] Van Der Spoel D, Lindahl E, Hess B, Groenhof G, Mark AE, Berendsen HJC. GROMACS: fast, flexible, and free. *J. Comput. Chem* 2005, 26, 1701 – 1718.

[6] Schmid N, Eichenberger AP, Choutko A, Riniker S, Winger M, Mark AE, van Gunsteren WF. Definition and testing of the GROMOS force-field versions 54A7 and 54B7. *Eur Biophys J* 2001, 40(7), 843 – 856

[7] Hermans J, Berendsen HJC, van Gunsteren WF, Postma JPM. A Consistent Empirical Potential for Water-Protein Interactions. *Biopolymers* 1984, 23, 1513–1518.

[8] Malde AK, Zuo L, Breeze M, Stroet M, Poger D, Nair PC, Oostenbrink C, Mark AE. An Automated force field Topology Builder (ATB) and repository: version 1.0. *J Chem Theory Comput*, 2011, 7(12), 4026–4037.

[9] Hess B, Bekker H, Berendsen HJC, Fraaije JGEM. LINCS: A Linear Constraint Solver for Molecular Simulations. *J Comput Chem* 1997, 18, 1463–1472.

[10] Miyamoto S, Kollman PA. SETTLE: An Analytical Version of the Shake and Rattle Algorithm for Rigid Water Models, *J Comput Chem* 1992, 13, 952–962.

[11] Kabadi AM, Gersbach CA. Engineering synthetic TALE and CRISPR/Cas9 transcription factors for regulating gene expression. *Methods* 2014, 69, 188–197.

constructs used an inactivated form of *S. pyogenes* Cas9 protein harboring D10A and H840A mutation.

TALE was custom-designed by Genecopoeia TALE-TF service and fused with effector domain VP64 at -99 (TALE-99) and -128 (TALE-128). Each TALE targets two more nucleotides than its corresponding ZF (a total of 20 nt), designed to start at the nearest thymine nucleotide. Zinc finger (ZF) fused with effector domain VP64 at -97 (ZF-97) and -126 (ZF-126) were as described previously.[12]

pEf1a-GFP/mCherry-sgRNA (5.3kb) and large EGFP (10.3 kb) plasmid were provided by the Lister Lab (UWA, unpublished data). Briefly, both GFP and mcherry were driven by Ef1a promoter and contains short IVS sequence which enhances transcription of the fluorescent protein. The SV40 polyadenylation signal C-terminus of IVS prevents transcriptional read through into the sgRNA expression cassette. Large EGFP vector is part of a TALE-DNMT3A fusion expression vector. The EGFP cassette is situated downstream of the TALE fusion and is separated by a HSV polyadenylation signal. Similarly, this EGFP is also driven by an Ef1a promoter.

1.3.2 Gel retardation assays

Polymer solutions were made up to a final concentration of 10 mM primary amines in filtered milli-Q water (calculations based on elemental results). Solutions were mixed with plasmid DNA (pDNA) at various nitrogen-to-phosphorus (N/P) ratios and incubated at room temperature for 30 min. Binding studies were conducted in water without the presence of additional buffers. The samples were electrophoresed on 1% w/v agarose gels in sodium borate (SB) buffer, stained with ethidium bromide (EtBr). Images were taken using ChemiDoc MP Imaging System.

1.3.3 DLS and zeta potential optimisations

Polymer solutions were mixed with pDNA at appropriate N/P ratios and incubated at room temperature for 30 min. Size and zeta potential of the resulting polyplexes were characterised using dynamic light scattering (Zetasizer Nano ZS), using a 4 mW He-Ne laser operating at 633 nm with a scattering angle of 173°. Measurements were taken in triplicate after an initial equilibration period of 2 min. For calibration of the measurements 'material' was defined as PGMA (refractive index of 1.515 and absorbance of 0.05) and 'dispersant' was defined as water at 25 °C (refractive index of 1.330 and viscosity of 0.887). The intensity-weighted zeta potential and hydrodynamic radius of the polyplexes is presented as mean ± standard deviation. All zeta potential measurements were taken at pH ≈ 6.

1.4 Cell culture and gene transfection

HEK293T (human embryonic kidney cell line, ATCC) and HeLa (human cervical adenocarcinoma cell line) were cultured in Dulbecco's Modified Eagle's Medium (DMEM, Gibco) with 10% heat-inactivated Fetal Bovine Serum (FBS, Life Technologies) and 1x GlutaMAX (Gibco). MCF-7 (human breast adenocarcinoma cell line, ATCC) were cultured in Minimum Essential Medium α (MEM α, Gibco) supplemented with 1% sodium bicarbonate, 10% FBS and 1x GlutaMAX. All cell lines were grown in a humidified incubator at 37 ^{o}C with 5% CO_2. Cell lines were seeded in 12-well plates

[12] Beltran A, Parikh S, Liu Y, Cuevas BD, Johnson GL, Futscher BW, Blancafort P. Re-activation of a dormant tumor suppressor gene maspin by designed transcription factors. *Oncogene* 2007, 26, 2791–2798.

(Corning Costar, Sigma Aldrich). Cell seeding densities are summarised in Supplementary Table 3. No antibiotic/antimycotic was used.

Supplementary Table 3: Seeding densities of cell lines used for transfected experiments.

Cell line	Seeding Density (cells/well)
HEK293T	2.0×10^5
MCF-7	1.4×10^5
HeLa	1.8×10^5

For transfection, polymer solution and pDNA were diluted to the required concentration in Opti-MEM reduced serum media (Gibco). For cotransfection experiments, pDNAs were mixed at a 1:1 ratio based on mass. pDNA solution was added to polymer solution (order of addition important) and mixed thoroughly so as to achieve the optimal N/P ratio for 1 µg DNA in total volume of 130 µL, and incubated at room temperature for 30 min. Commercial transfection agent Lipofectamine 2000 (Invitrogen) was used as a standard according to the manufacturer's protocol. 130 µL of polyplex solution was transferred to the appropriate well containing 300 µL of Opti-MEM. After a 4 h incubation period, an additional 570 µL of the appropriate complete culture medium was added and experiment was incubated for a further 44 h, giving a total transfection time of 48 h.

Transfection efficiency was observed using fluorescent microscopy and quantified via flow cytometry. Fluorescent and phase contrast images were taken using an Olympus IX-71 inverted microscope (U-MGFPHQ and U-MRFPHQ filters). Flow cytometry of EGFP transfections were conducted using BD FACSCantoII flow cytometer, cotransfection experiments were analysed using LSRII Fortessa flow cytometer. In preparation for flow cytometry, cells were washed twice with 1 X phosphate buffered saline (PBS) and harvested with trypsin (Life Technologies). Cells were collected via centrifugation (300*g*, 5 min) and washed in 500 µL FACS buffer (2% FBS, 4 mM EDTA in PBS). Cells were centrifuged again (300*g*, 5 min) and resuspended in 200 µL of FACS buffer for flow analysis. Samples were acquired using BD FACS Diva software. EGFP was excited by 488 nm laser, and emission was measured with 502 nm long pass and 530/30 nm band pass filters; RFP was excited by 561 nm laser, and emission was measured with 600 nm long pass and 610/20 nm band pass filters. 30,000 single cell events, gated on forward scatter area vs height, were recorded for analysis. Post-acquisition analysis was performed on FlowJo vX software.

1.5 Cytotoxicity

Cytotoxicity was quantified by cell viability following 24 or 48 h incubation of cells with transfection cocktails. HEK293T, HeLa, or MCF-7 cells were seeded in poly-L-lysine (Sigma) treated 96-well opaque white plates (Corning) at densities as shown in Supplementary Table 4.

Supplementary Table 4: Seeding density and mass of pDNA used for cytotoxicity experiments.

Cell line	Seeding Density (cells/well)	pDNA (ng/well)
HEK293T	1.65×10^4	84
MCF-7	1.18×10^4	84
HeLa	1.35×10^4	84

Cells were allowed to settle for 24 h, the media was removed, cells were washed with PBS once,

and Opti-MEM media containing transfection cocktails without serum were applied. After 4 h, media was replaced with complete media containing serum. Cells were left for a further 20 or 44 h, and CellTiter-Glo reagent (Promega, 10 µl per well) was added. Plates were shaken and incubated for times according to the manufacturer's protocol and then read on a luminescence plate reader (PerkinElmer EnSpire series). Measured values were normalised to control wells, which were treated identically, but received Opti-MEM containing no transfection agents. All conditions were measured at least in triplicate. Additional controls (pDNA only, unwashed wells) were measured to confirm no toxicity of the plasmid alone, loss of viability owing to Opti-MEM treatment, or loss of cells due to washing.

1.6 Functional outcome transfection study

MCF-7 cells were transfected with CRISPR/dCas9, TALEs and zinc fingers, using the transfection protocol above. The plasmids were delivered in mixtures as summarised in Supplementary Table 5. Phase contrast images were taken for all the conditions prior to RNA extraction. TALE plasmids incorporated an EGFP sequence and were imaged with a fluorescent microscope as described previously.

Supplementary Table 5: Plasmid constructs and mixture ratios used for transfection experiments.

**Construct 1**	**Construct 2**	**Mass Ratio (1 : 2)**
CRISPR/dCas9-VP64	pSPgRNA (mix of 4)	2 : 1
TALE_99-VP64	TALE_128-VP64	1 : 1
ZF_99-VP64	ZF_126-VP64	1 : 1

1.6.1 mRNA extraction and reverse-transcription PCR

Total RNA was extracted using 1 ml Trizol reagent (Invitrogen) for approximately 1×10^7 cells, according to the manufacturer's protocol. Extracted total RNA was converted to cDNA using reverse transcriptase-PCR using the High Capacity cDNA Reverse Transcription Kit (4368813, Applied Biosystems).The expression levels of MASPIN were analysed with real time reverse transcription quantitative PCR (RT-qPCR) with GAPDH as housekeeping control. Primer sequences used to detect mRNA levels were commercially purchased: MASPIN (Hs Hs00985285_m1, Thermo Fisher), GAPDH (4332649, Applied Biosystems). The MultiScribe Reverse Transcriptase (Life Technologies) was used to synthesise cDNA from extracted total RNA with commercially available gene specific primers, and quantitative PCR was carried out using TaqMan Universal PCR Master Mix (Life Technologies). Analysis was performed in Rotor Gene Q (Applied Biosystems) using TaqMan Fast Universal PCR Master Mix (4352042, Applied Biosystems).

1.6.2 Immunoblotting

Cells were lysed with lysis buffer (50 mM Tris–HCl, 150 mM NaCl, 1%NP40, 0.5% sodium deoxycholate, 1% Triton 100, 50 mN NaF, 5 mM $NaVO_3$ and 100 mM PMSF) 48 hours post-transfected as previously described.[13] Total cell lysates were separated by SDS-PAGE and then

[13] Plani-Lam JH, Chow TC, Siu KL, Chau WH, Ng MH, Bao S, Ng CT, Sham P, Shum DK, Ingley E, Jin DY, Song YQ. PTPN21 exerts pro-neuronal survival and neuritic elongation via ErbB4/NRG3 signaling. *Int J Biochem Cell Biol* 2015, 61, 53–62.

transferred to PVDF membranes (Applied Biosystems) and visualised using Odyssey (LI-COR, Bioscience). Antibodies used to detect the protein levels were MASPIN (554292, BD Biosciences) and α-Tubulin (T6199, Sigma). MCF-7 cells were transfected with CRISPR/dCas9, TALEs or zinc fingers, using the transfection protocol above (section 1.4). The plasmids were delivered in mixtures as summarised in Supplementary Table 5. Brightfield images were taken for all the conditions prior to RNA extraction. TALE plasmids incorporated an EGFP sequence and so were imaged with a fluorescent microscope as described previously.

1.7 Statistical Analysis

Statistical analyses were performed with Graphpad Prism (GraphPad Software Inc.) The data is illustrated as the average, error bars represent the standard deviation. For all tests, differences were considered significant at $p \leq 0.05$ (*), $p \leq 0.01$ (**), $p \leq 0.001$ (***), $p \leq 0.0001$ (****). Unless otherwise specified, data are represented as the means of technical triplicate and biological duplicate independent experiments.

2 Supplemental Data

2.1 Polymer backbone characterisation

2.1.1 ^{1}H NMR spectra of polymer backbones

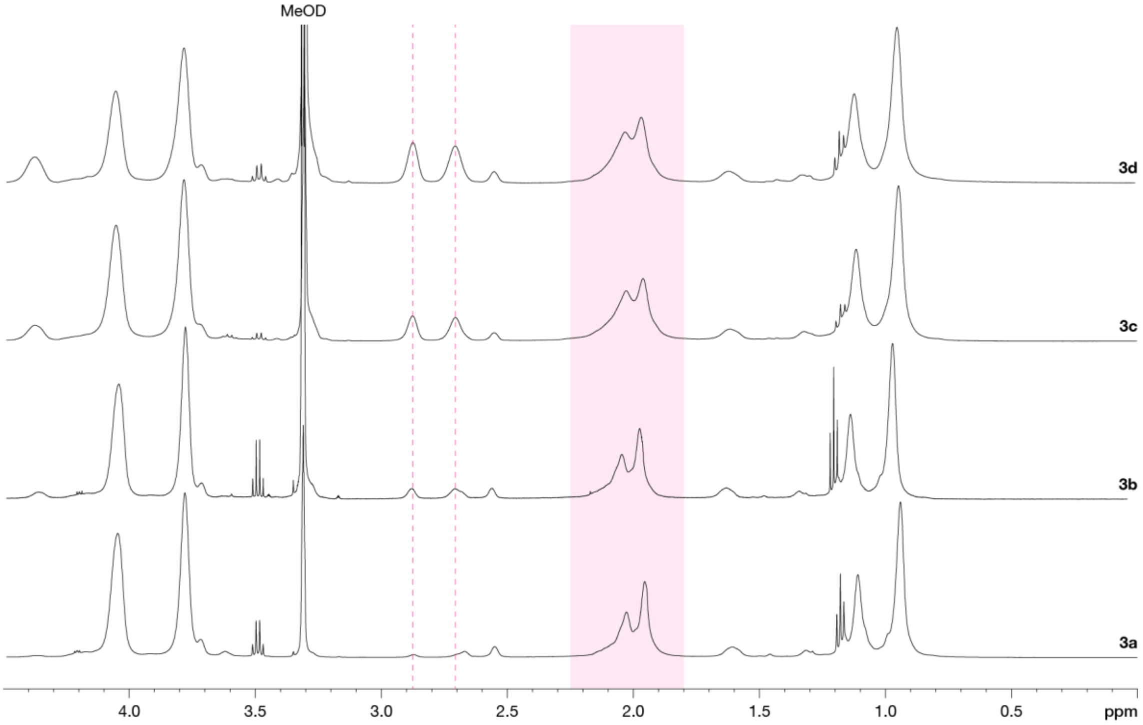

Supplementary Figure 1: ^{1}H NMR spectra of copolymer backbones. Spectra were taken in methanol-d4 at 500 MHz. Peak corresponding to the epoxide moiety on glycidyl methacrylate (δ 2.7, 2.9 ppm) was used to calculate GMA incorporation for copolymer composition, against the backbone methylene bridge (shaded).

2.1.2 Monomer reactivity ratios

The molar composition of each polymer backbone was determined by ^{1}H NMR. M_1 refers to the mole fraction of monomer **1** in the reaction system while m_1 refers to the mole fraction of monomer **1** in the polymer. Relationship is plotted as such:

$$\frac{M_1(m_2 - m_1)}{M_2 m_1} = \left(-\frac{m_2 M_1^2}{m_1 M_2^2}\right) r_1 + r_2$$

where r_1 and r_2 are the reactivity ratios of monomers **1** and **2**, and are given as the gradient and intercept. Experimental values for the reactivity ratios of monomers HEMA and GMA are 1.07 and 0.729 respectively; since $r_1 \approx r_2 \approx 1$ this indicates a random statistical copolymer structure. [Method from Tidwell & Mortimer[14]]

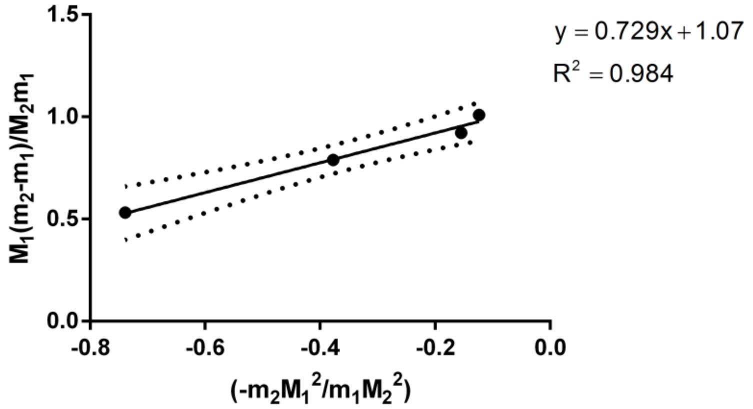

Supplementary Figure 2: Experimental determination of the reactivity ratios of monomers glycidyl methacrylate (GMA) and hydroxyethyl methacrylate (HEMA), using the Linearisation (Fineman and Ross) method. Line of best fit is shown with 95% confidence interval.

[14] Tidwell PW, Mortimer GA. An improved method of calculating copolymerization reactivity ratios. *Polymer Chem* 1965, 3(1), 369–387.

2.2 Dendronised polymer characterisation

2.2.1 Generation 4 dendronised polymer

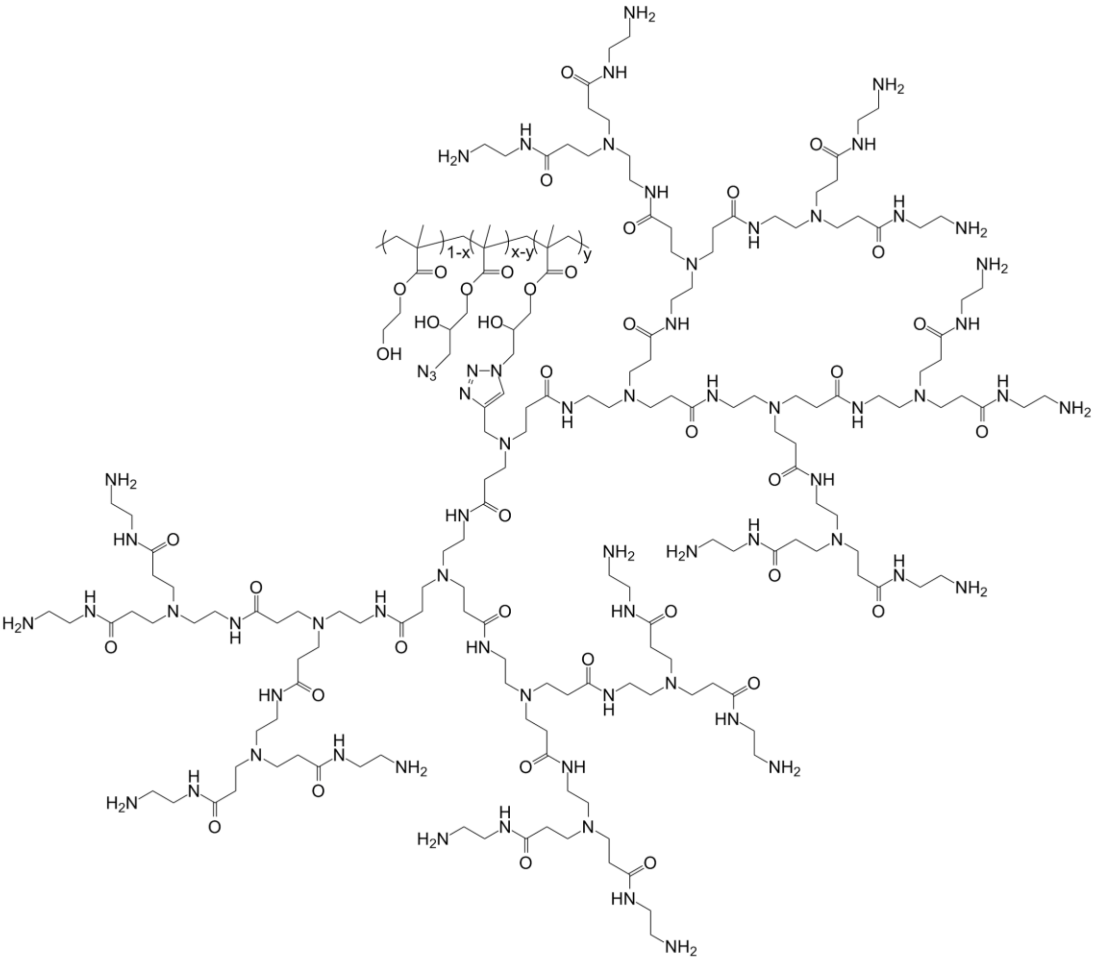

Supplementary Table 6: Experimental elemental analysis of fourth generation dendron-functionalised copolymers.

Polymer	Monomer Ratio (1-x : x-y : x)	% C	% H	% N	% F
**9a**	32.2 : 0 : 1	50.75	8.06	12.21	-
		50.58	7.91	12.17	-
**9b**	11.5 : 0 : 1	42.10; 42.07	7.74; 7.82	14.28; 14.34	-
		48.98; 49.11 **(50.46)***	7.96; 7.85	16.32; 16.39 **(16.51)***	-
**9c**	4.88 : 0 : 1	50.43	8.78	20.86	-
		50.27	8.71	20.89	-
**9d**	12 : 1 : 3.76	50.47	8.77	21.62	-
		50.24	8.81	21.57	-

***** Corrected for water content – sample was hygroscopic, measurements were conducted twice in duplicate with repeated drying, before being normalised to average carbon percentage.

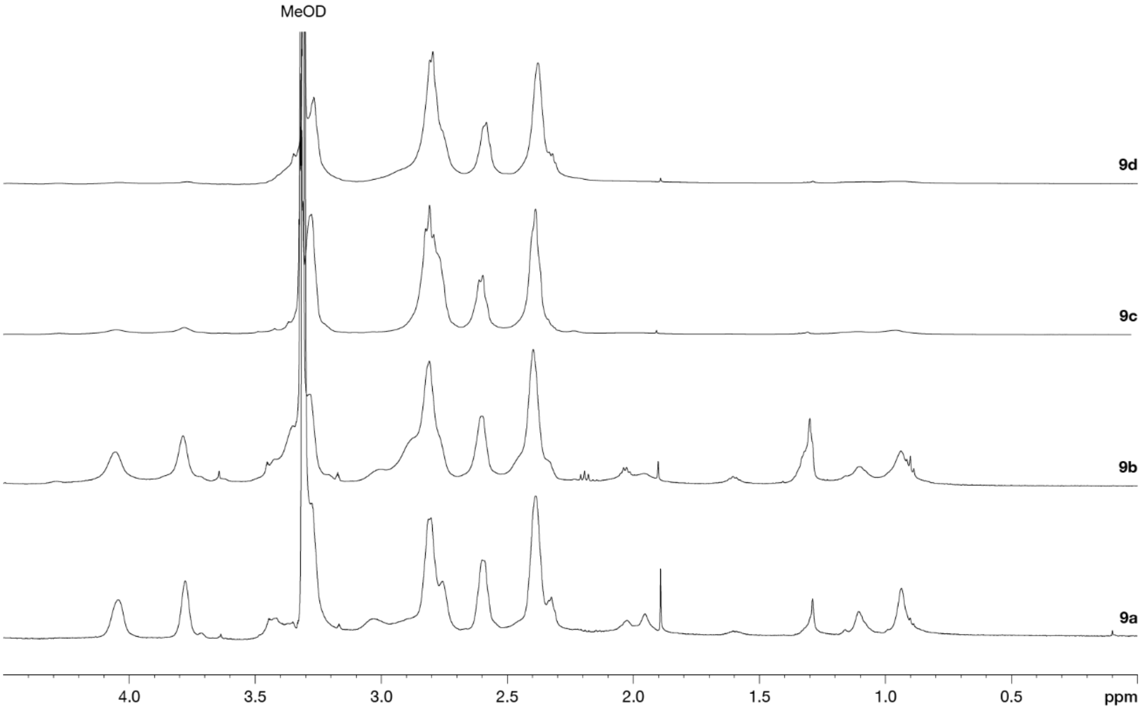

Supplementary Figure 3: ^{1}H NMR spectra of polymers with 4th generation PAMAM dendrons attached. ^{1}H NMR spectra were taken in methanol-d4 at 500 MHz. Peaks corresponding to backbone disappear as dendron substitution increases.

2.2.2 Generation 5 dendronised polymer

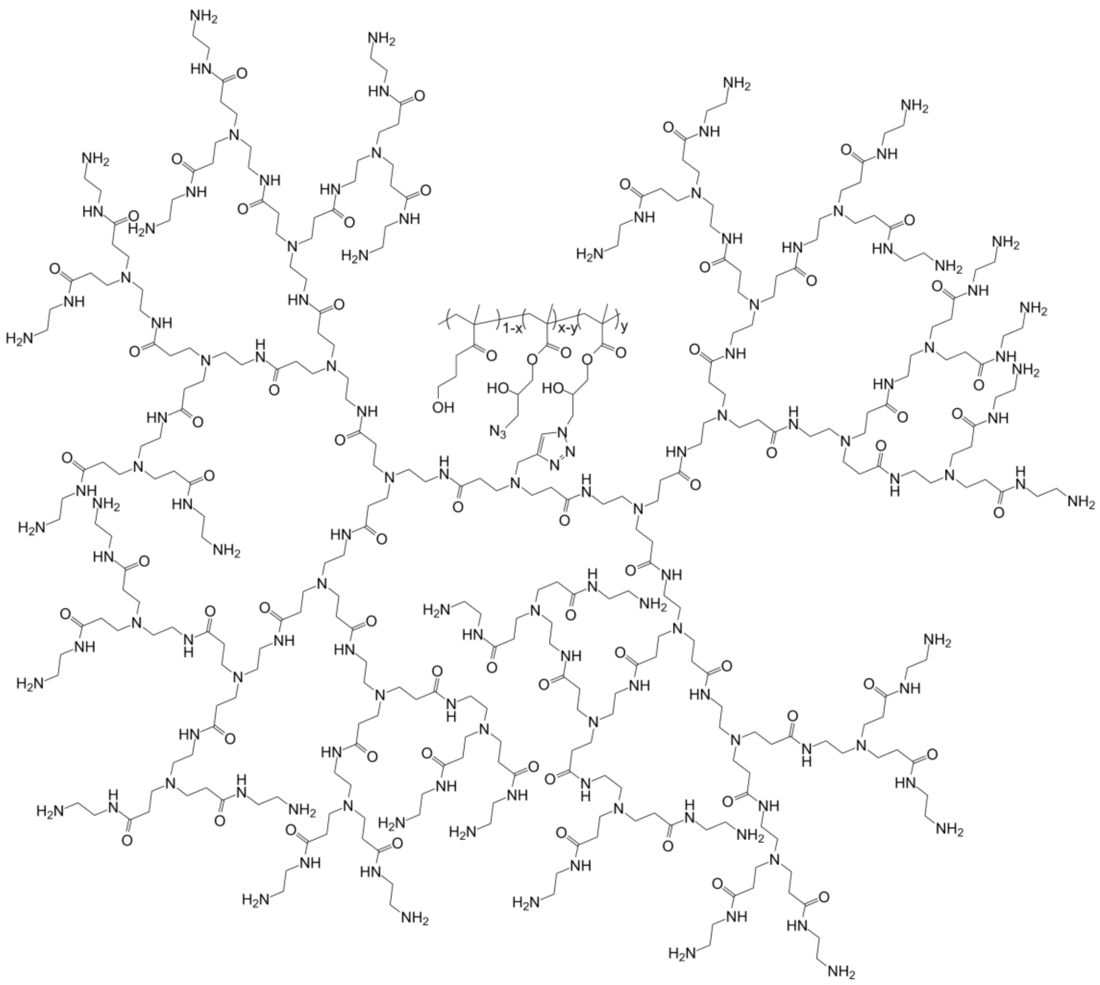

Supplementary Table 7: Experimental elemental analysis of fifth generation dendron-functionalised copolymers.

Polymer	Monomer Ratio (1-x : x-y : y)	% C	% H	% N	% F
**10a**	32.2 : 0 : 1	50.86	8.22	15.09	-
		50.63	8.25	15.02	-
**10b**	11.5 : 0 : 1	40.60; 40.54	7.90; 7.95	15.54; 15.44	-
		45.14; 45.84 **(50.36)***	7.99; 8.21	17.18; 17.48 **(19.14)***	-
**10c**	16.6 : 1 : 2.4	50.11	8.90	21.62	-
		50.37	8.96	21.68	-
**10d**	5.54 : 1.15 : 1	50.23	9.00	22.05	-
		49.94	8.82	21.91	-

***** Corrected for water content – sample was hygroscopic, measurements were conducted twice in duplicate with repeated drying, before being normalised to average carbon percentage.

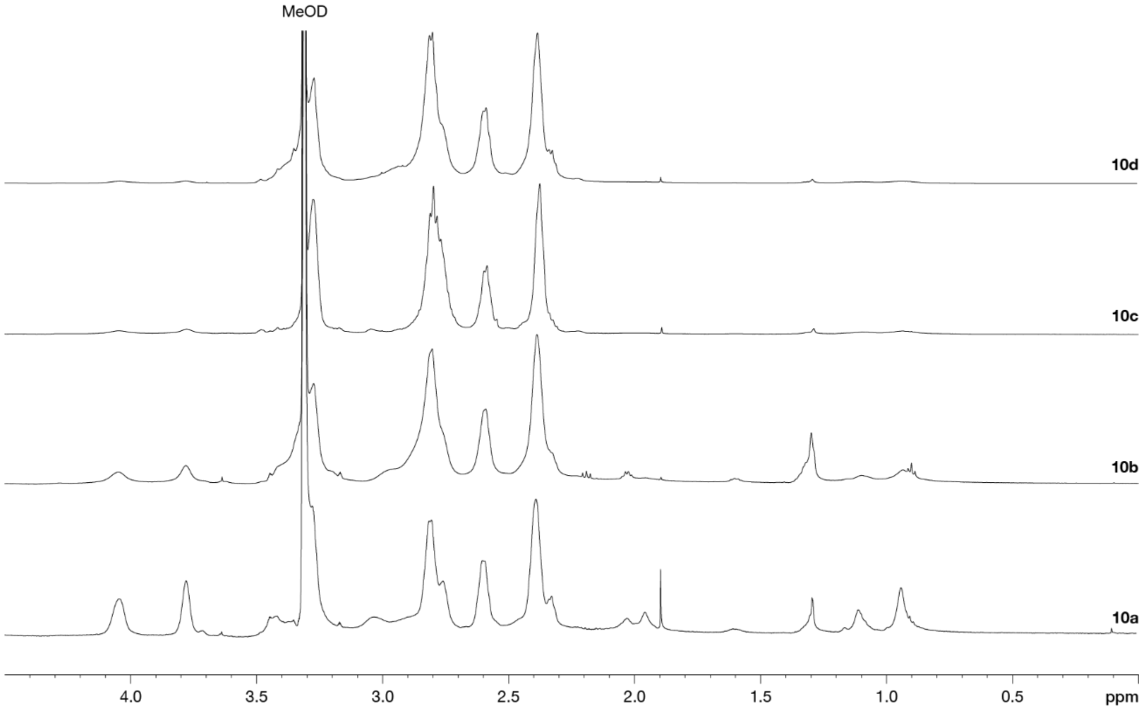

Supplementary Figure 4: ^{1}H NMR spectra of polymers with 5th generation dendrons attached. ^{1}H NMR spectra were taken in methanol-d4 at 500 MHz. Peaks corresponding to polymer backbone disappear as dendron substitution increases. **a**, 3% (**10a**); **b**, 8% (**10b**); **c**, 17% (**10c**); **d**, 28% (**10d**).

2.2.3 IR spectra

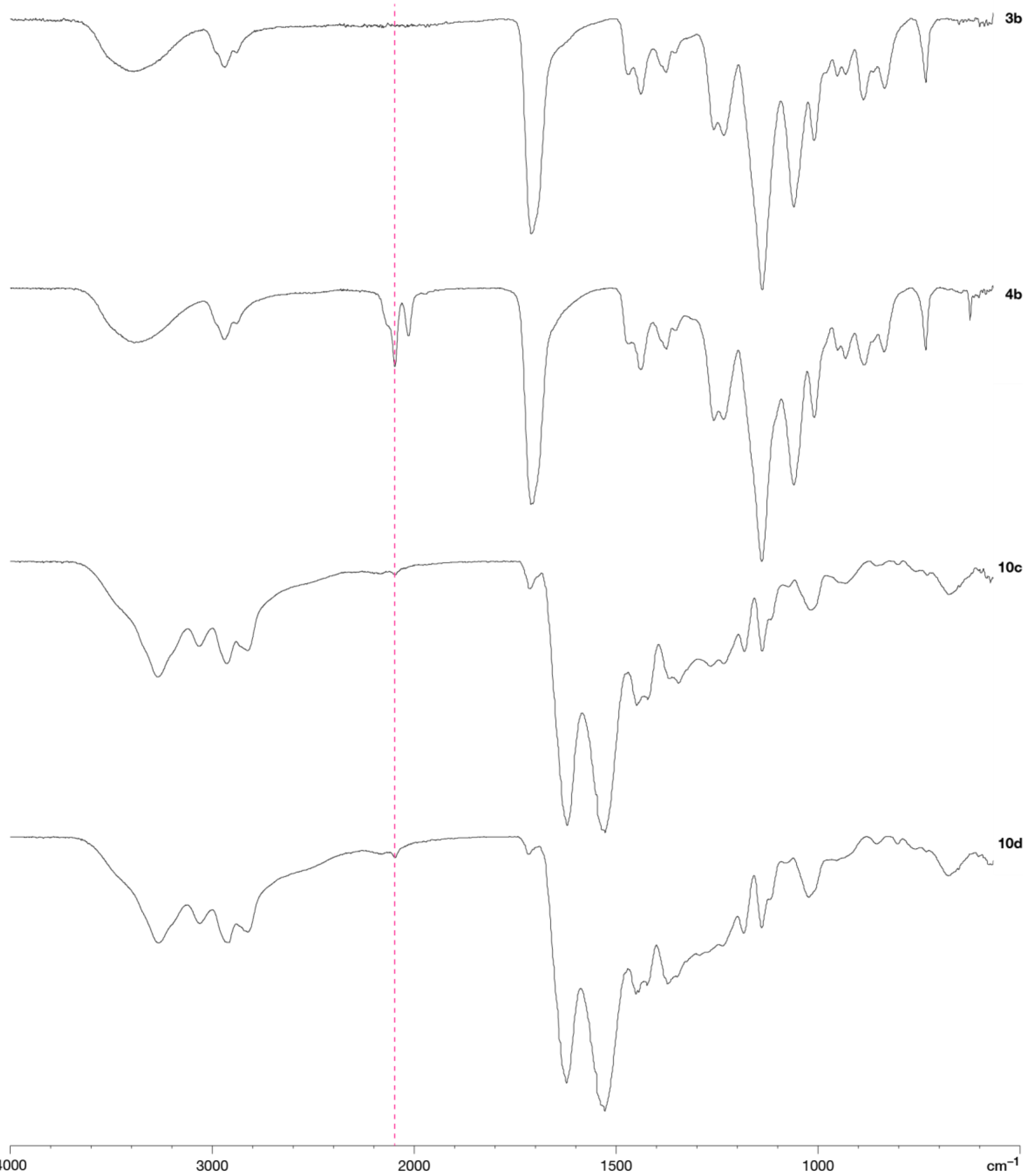

Supplementary Figure 5: FTIR spectra of polymers demonstrating unreacted azide residues in high GMA composition copolymers. Dendron substitution in polymers with high GMA composition did not react to completion due to steric hindrance of dendrons, as confirmed by residual azide peak at 2105 cm^{-1}. **3b**, Representative polymer backbone. **4b**, Representative azide functionalised copolymer. **10c**, **10d**, Representative dendronised polymers showing incomplete click reaction and presence of unreacted azide (dotted line).

2.2.4 Substitution Pattern

Supplementary Table 8: Dendron substitution along copolymer backbone, given as a percentage of reacted epoxide moieties from GMA.

		Dendron Generation				
		**1**	**2**	**3**	**4**	**5**
**Substitution (%)**	**3**	100	100	100	100	100
	**8**	100	100	100	100	100
	**17**	-	-	100	100	67
	**28**	-	-	75	75	45

2.2.5 Molecular Weight

Supplementary Table 9: Polymer molecular weights (kDa) as calculated from elemental results and original backbone measurement (GPC).

		GMA (mol %)			
		**3**	**8**	**17**	**28**
**Dendron Generation**	**1**	16.2	18.6	-	-
	**2**	17.8	23.3	-	-
	**3**	20.9	32.6	72.8	66.8
	**4**	28.4	53.5	123.9	117.1
	**5**	41.4	90.7	145.3	143.0

2.3 Optimisation of transfection: systematic analysis of backbone and dendron generation

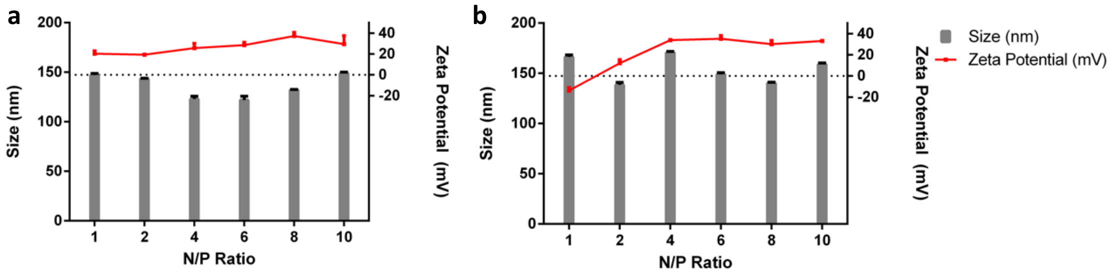

Supplementary Figure 6: DLS and Zeta polyplex optimisation for poly(amido amine) (PAMAM) dendrimer (**a**) and **10c** polymer (**b**), using 5.3 kb EGFP plasmid. Binding was assessed in milli-Q water at pH ≈ 6 without additional buffers. All plasmid is bound at N/P ratio of 1 in the case of the PAMAM dendrimer, while the **10c** polymer formulation only binds all pDNA at or above an N/P ratio of 2. Resulting bound polyplexes are of similar size and zeta potential for both PAMAM and **10c** at all higher N/P ratios.

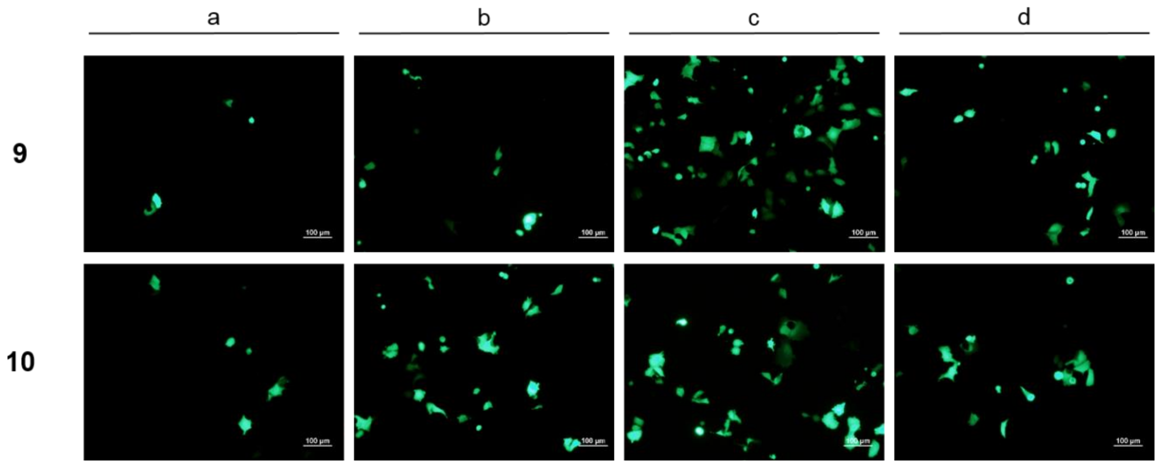

Supplementary Figure 7: EGFP expression representing successful transfection in MCF-7 cell line at N/P ratio of 5. Images are representative of technical duplicates and biological duplicates.

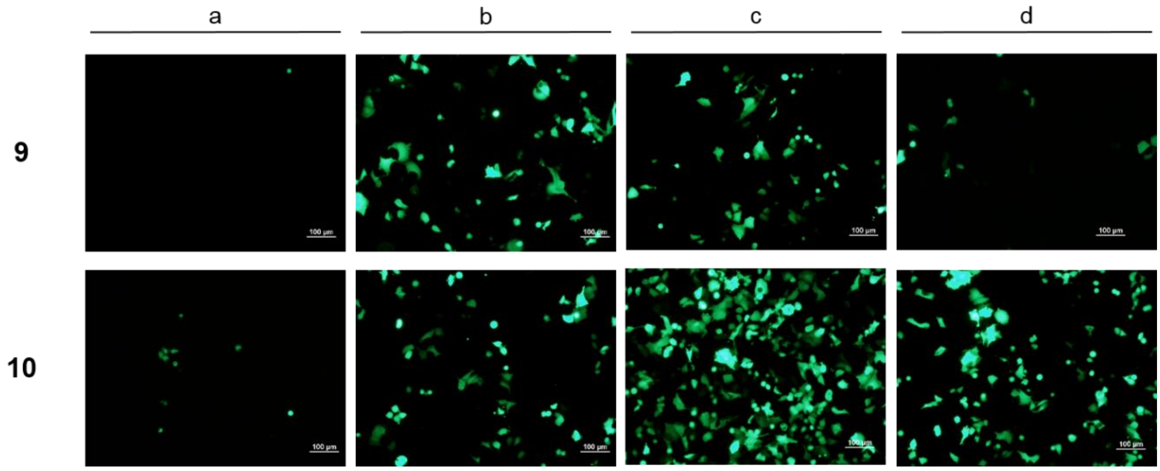

Supplementary Figure 8: EGFP expression representing successful transfection in MCF-7 cell line at N/P ratio of 10. Images are representative of technical duplicates and biological duplicates.

2.3.1 Cytotoxicity

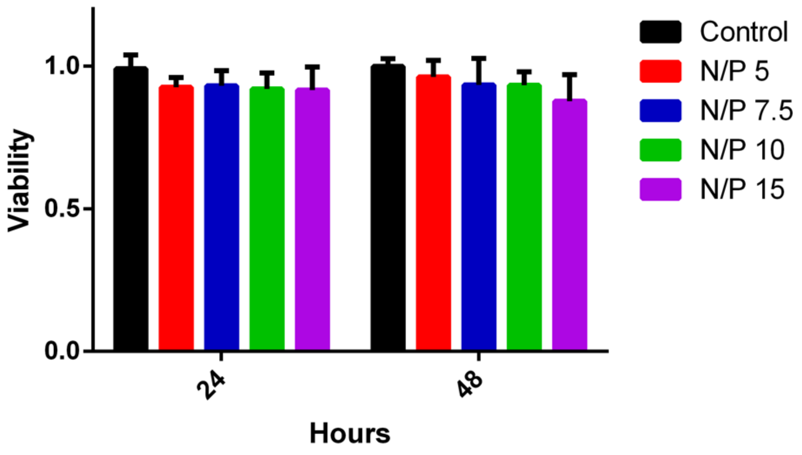

Supplementary Figure 9: Cell viability for MCF-7 cell line when transfected using **10c** polymer formulation. Cells were transfected with 5.3 kb EGFP plasmid at various N/P ratios and cell viability was assessed at 24 and 48 h. Culture conditions were identical to those used in the transfection experiments. There was no significant difference relative to control ($p > 0.05$) observed in cell viability up to the highest concentration tested (N/P 15).

2.4 Molecular dynamics analysis

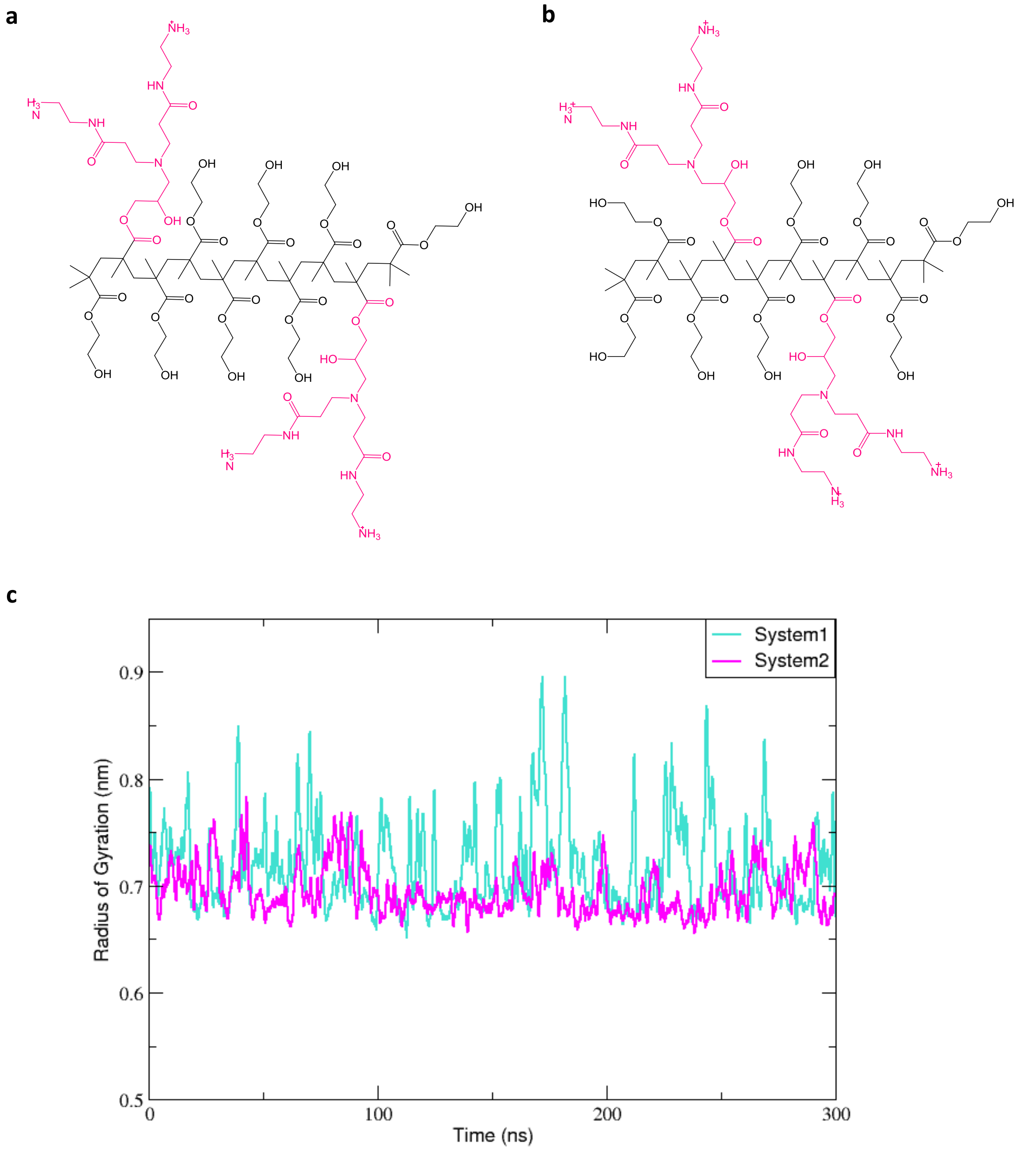

Supplementary Figure 10: Model systems used for molecular dynamics simulations and radius of gyration of polymer systems calculated for 300 ns MD simulation. Radius of gyration, **c**, of System 1 (**a**, dendrons far apart) fluctuated over a greater range (nm) than that measured for System 2 (**b**, dendrons close together), indicating a higher degree of movement in System 1.

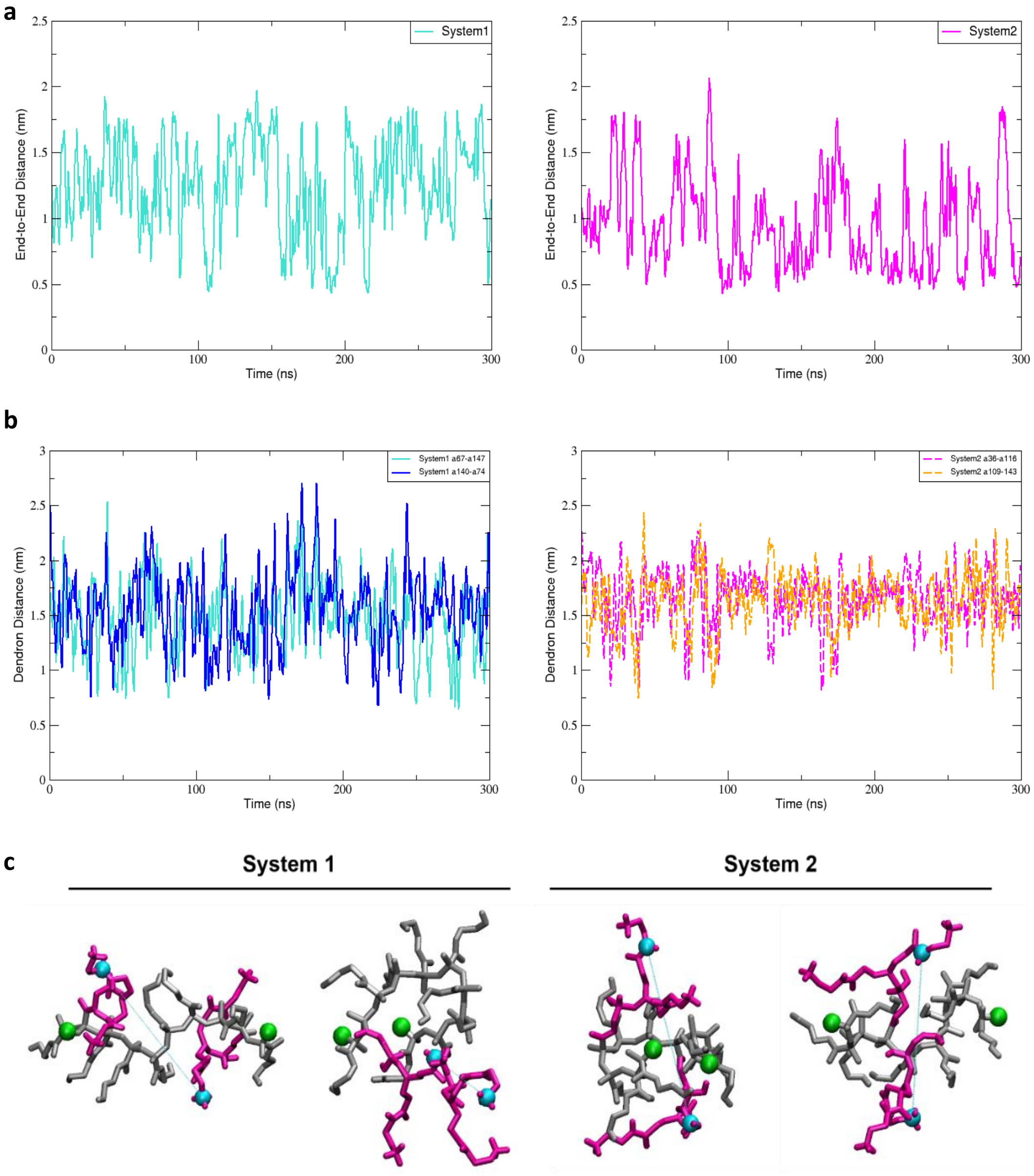

Supplementary Figure 11: Flexibility and conformation of polymers by point-to-point distance measurements. **a**, Distance between endpoints along backbone are measured (highlighted in green in **c**). End-to-end distance fluctuates rather than converging on a particular structure. However, System 1 fluctuates with a greater deviation than System 2, indicating higher flexibility. System 2 repeatedly returns to a structure where polymer ends at ≈0.7 nm apart. **b**, Distance between dendron branches were measured for the two systems (points shown in blue in **c**). Graphs demonstrate again that System 1 has greater movement that System 2, which repeatedly returns to a conformation where the dendrons are ≈1.7 nm apart. **c**, Snapshots representative of the conformations taken by each system. HEMA backbone is represented in grey, dendrons in pink. System 1 fluctuated between two extreme conformations, while System 2 preferentially remains in a conformation where the dendrons are on either side of the backbone, while backbone endpoints (green) fluctuate between a stretched linear, and wrapped conformations.

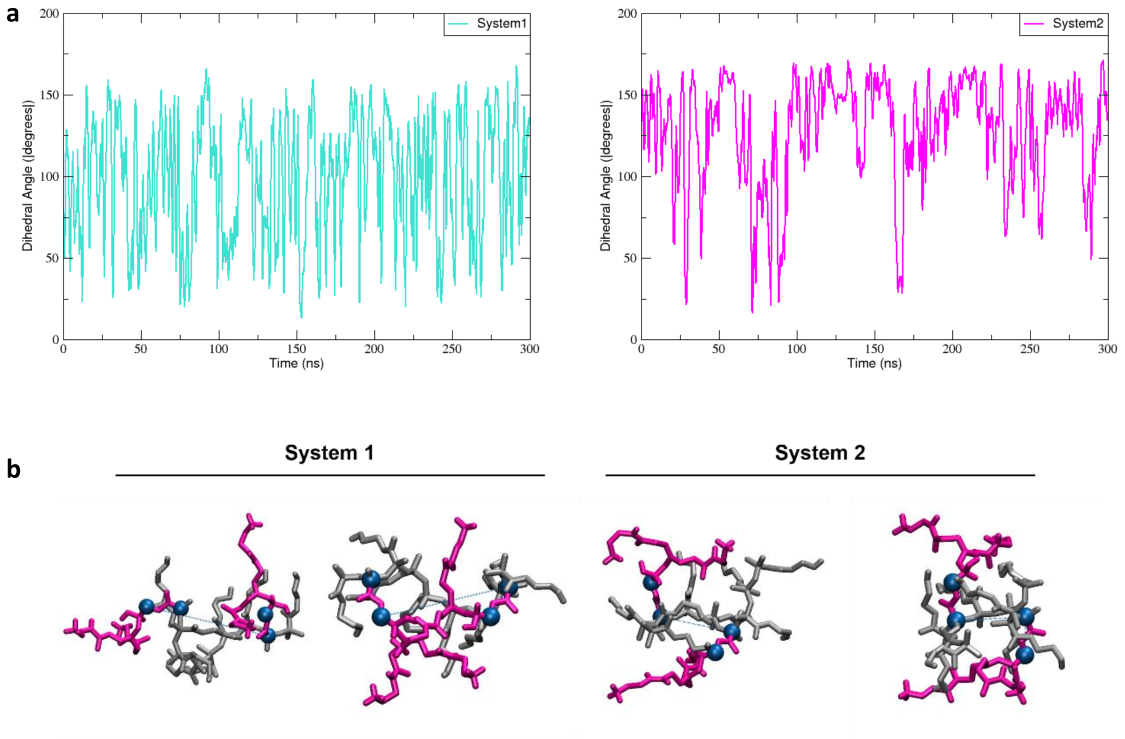

Supplementary Figure 12: Dihedral angle measurements of dendrons with time for Systems 1 and 2. Dihedral angle between the dendrons was defined as shown by the blue spheres, and the angle measured against simulation time. **a**, System 1 demonstrates high level of fluctuation, with angles moving frequently between ≈25° and ≈150°, with representative snapshots shown in **b**. System 2 demonstrates a preference to have a dihedral angle range of ≈125° to ≈170°; representative snapshots shown that this preference corresponds to conformations where the dendrons are on either side of the dendron, directed away from the other. HEMA backbone is represented in grey, dendrons in pink.

2.5 Fluorination of dendronised polymers

2.5.1 Elemental analysis

Supplementary Table 10: Experimental elemental analysis of fluorinated dendronised polymers.

Polymer	Dendron Generation	% C	% H	% N	% F
**12a**	5	44.11	6.43	11.45	14.02
		43.93	6.40	11.38	13.87
**12b**	5	40.00	5.64	12.03	21.65
		40.02	5.56	12.03	21.39
**11c**	4	41.61	5.64	13.59	20.00
		41.80	5.77	13.63	19.89
**12c**	5	39.74	5.43	12.60	23.10
		39.79	5.28	12.60	23.14
**12d**	5	43.97	6.47	14.72	16.50
		44.10	6.41	14.43	16.10

2.5.2 Fluorination percentage and molecular weights

Supplementary Table 11: Fluorination percentage and resulting molecular weight calculated from elemental results and original GPC measurements.

Polymer	Primary amines reacted (%)	Mw (kDa)
**12a**	39	63.4
**12b**	47	141.7
**11c**	40	145.6
**12c**	45	194.1
**12d**	31	214.5

2.6 Optimisation of transfection for small (5.3 kb) plasmid

2.6.1 Gel retardation assay

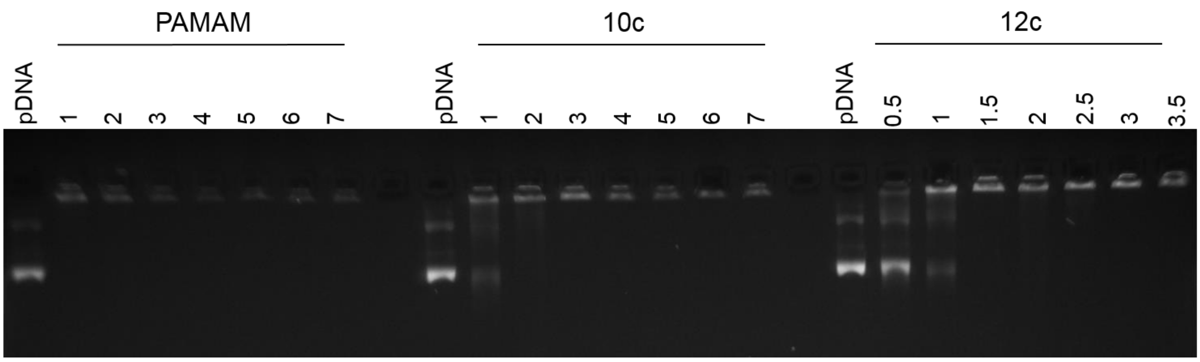

Supplementary Figure 13: Gel retardation assays conducted with 5.3 kb-sized pDNA. Gel demonstrates complete plasmid binding by N/P ratios of 1, 2 and 1.5 for the PAMAM dendrimer, **10c** polymer and **12c** polymer formulations respectively. pDNA without polymer was used as a control to assess retardation.

2.6.2 DLS/zeta optimisation

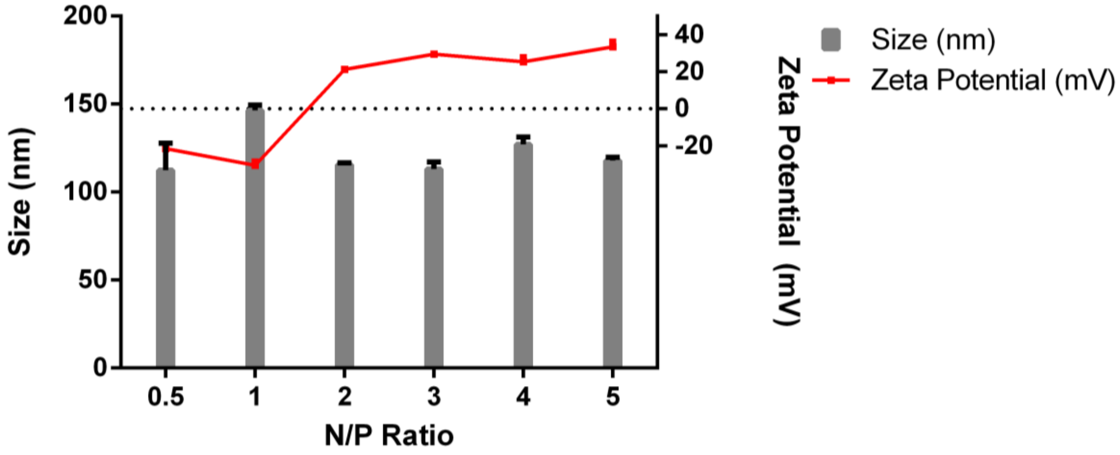

Supplementary Figure 14: DLS and Zeta optimisation of polymer **12c** with 5.3 kb plasmid. Polymer **12c** formulation bound all pDNA above an N/P ratio of 1. The size and zeta potentials of polyplexes resulting from fully-bound pDNA are similar to those demonstrated by PAMAM and polymer **10c**.

2.6.3 Representative transfection images (MCF-7)

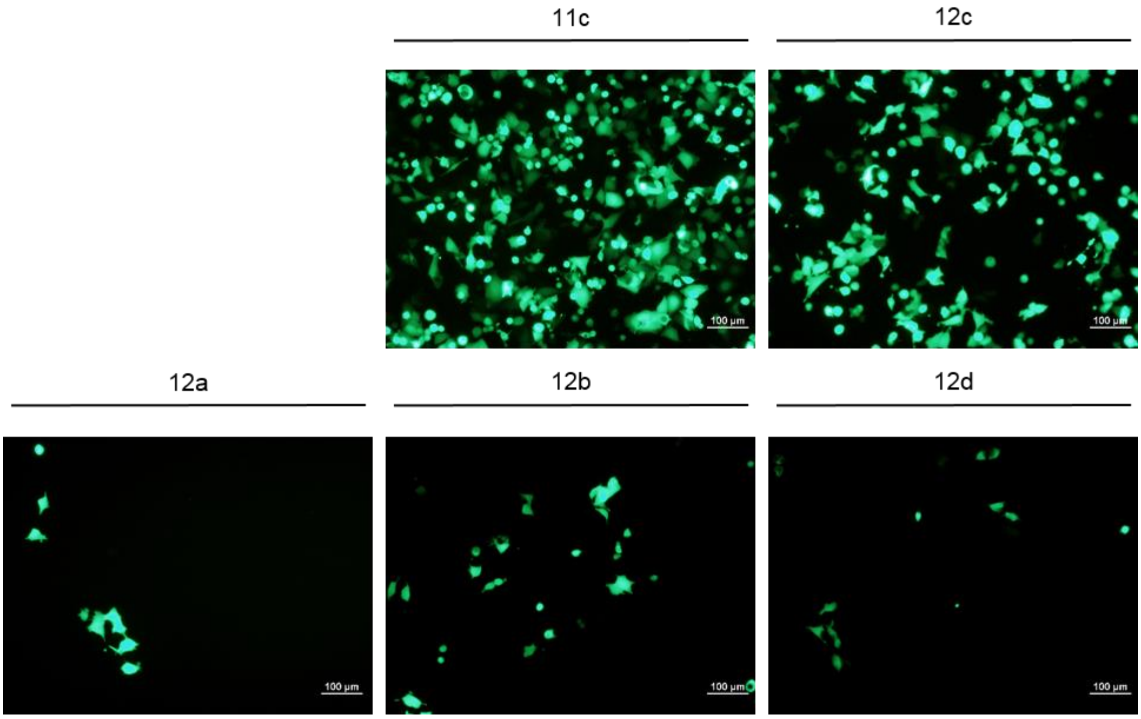

Supplementary Figure 15: EGFP expression representing successful transfection in MCF-7 cell line at N/P ratio of 2.5. Images are representative of technical duplicates and biological duplicates.

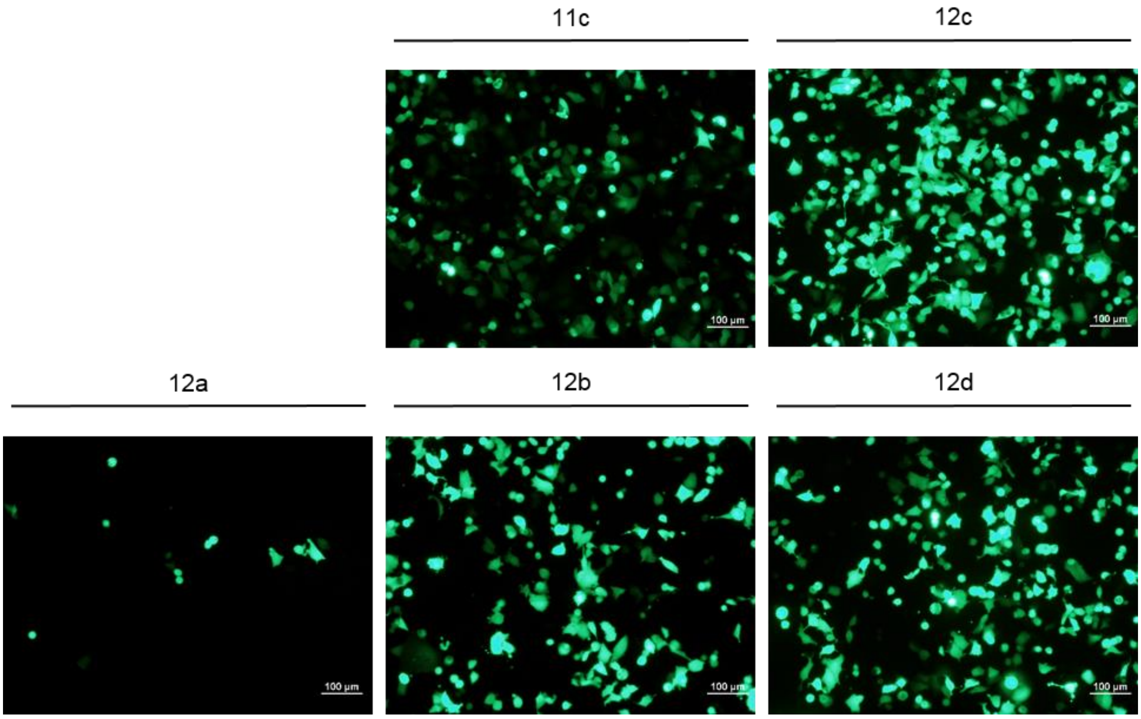

Supplementary Figure 16: EGFP expression representing successful transfection in MCF-7 cell line at N/P ratio of 5. Images are representative of technical duplicates and biological duplicates.

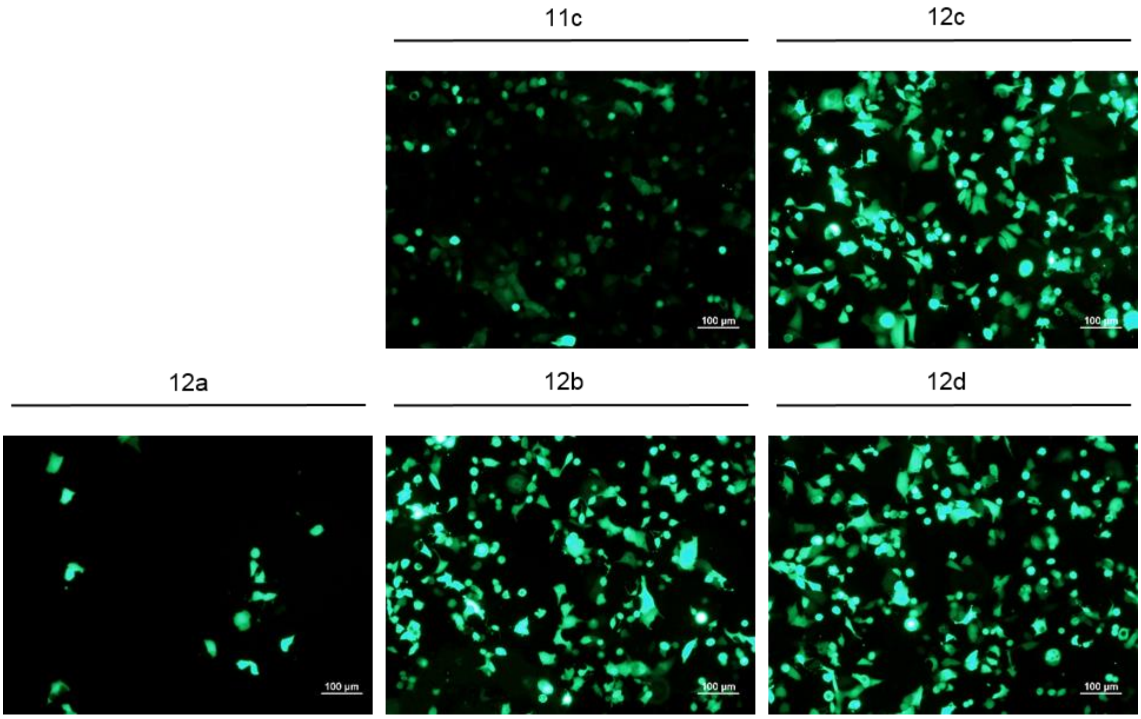

Supplementary Figure 17: EGFP expression representing successful transfection in MCF-7 cell line at NP ratio of 7.5. Images are representative of technical duplicates and biological duplicates.

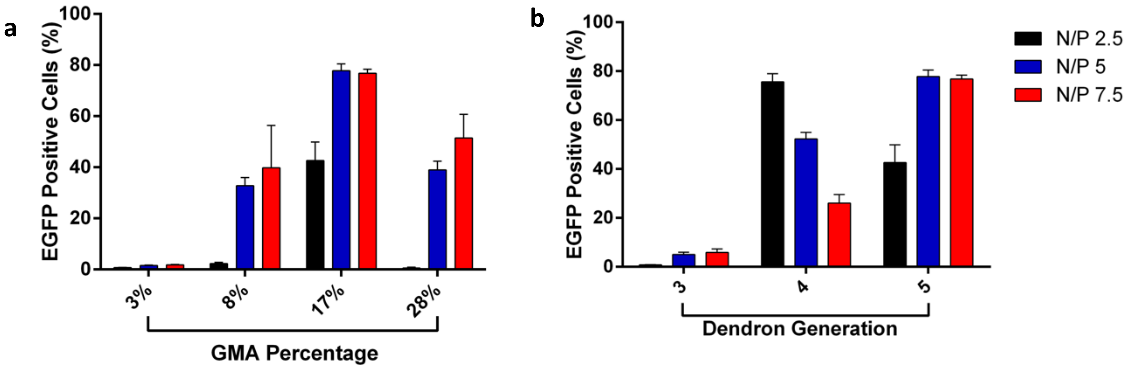

Supplementary Figure 18: Screening fluorinated polymer formulations for optimal transfection conditions. **a**, Polymer formulations with generation 5 fluorinated dendrons, and **b**, 'c' polymer formulation with generation 3 to 5 dendrons were screened at various N/P ratios to find optimal conditions.

2.6.4 Cytotoxicity

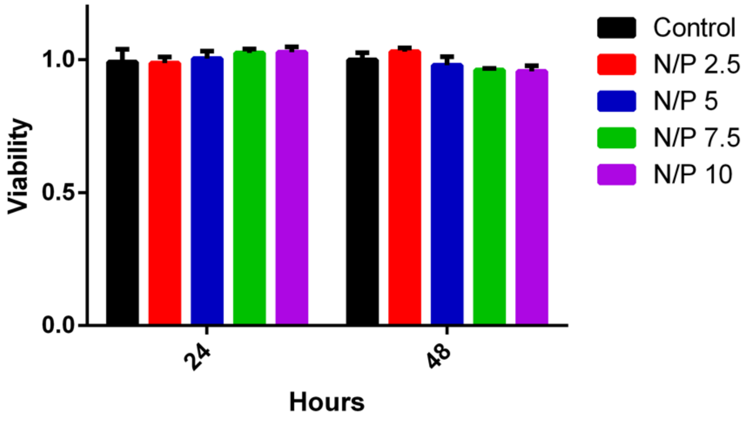

Supplementary Figure 19: Cell viability for MCF-7 cell line when transfected using **12c** polymer formulation. Cells were transfected with 5.3 kb EGFP plasmid at various N/P ratios and cell viability was assessed at 24 and 48 h. Culture conditions were identical to those used in the transfection experiments. There was no significant difference ($p > 0.05$) observed in cell viability up to the highest concentration tested (N/P 10).

2.7 Optimisation of transfection for large (10.3 kb) plasmid

2.7.1 Gel retardation assay

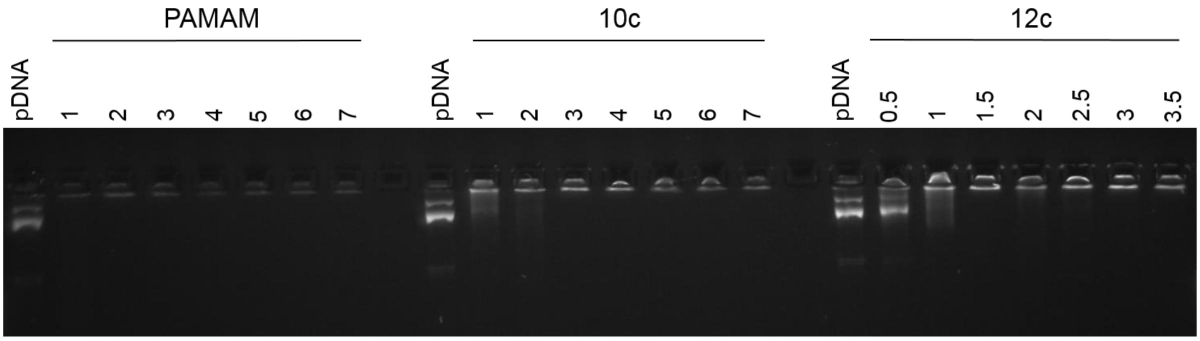

Supplementary Figure 20: Gel retardation assays conducted with 10.3 kb-sized pDNA. Gel demonstrates complete plasmid binding by N/P ratio of 1, 2 and 1.5 for the PAMAM dendrimer, **10c** polymer and **12c** polymer formulations respectively. pDNA without polymer was used as a control to assess retardation.

2.7.2 DLS/Zeta measurements

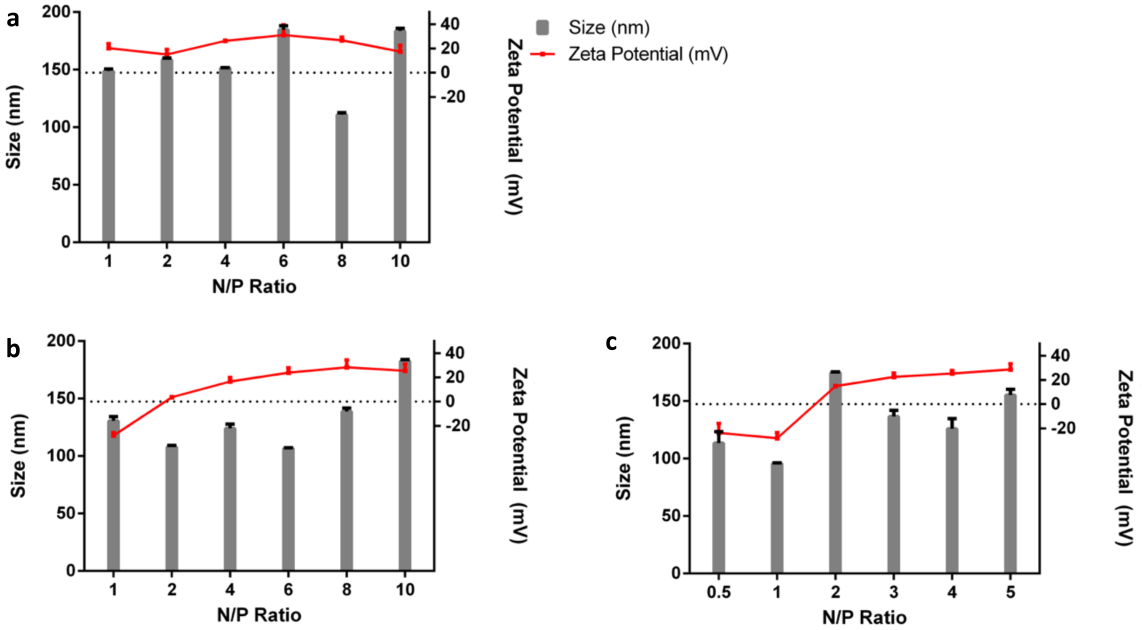

Supplementary Figure 21: DLS and Zeta polyplex optimisation for **a**, poly(amido amine) (PAMAM) dendrimer; **b**, polymer **10c**, and **c**, polymer **12c** using 10.3 kb EGFP plasmid. Binding was assessed in milli-Q water at pH ≈ 6 without additional buffers. All plasmid is bound at N/P ratio of 1 in the case of the PAMAM dendrimer, while the **10c** and **12c** polymer formulations only bind all pDNA at an N/P ratio of 2. Resulting bound polyplexes are of similar size and zeta potential for both PAMAM and polymer at all higher N/P ratios.

2.7.3 Representative transfection images

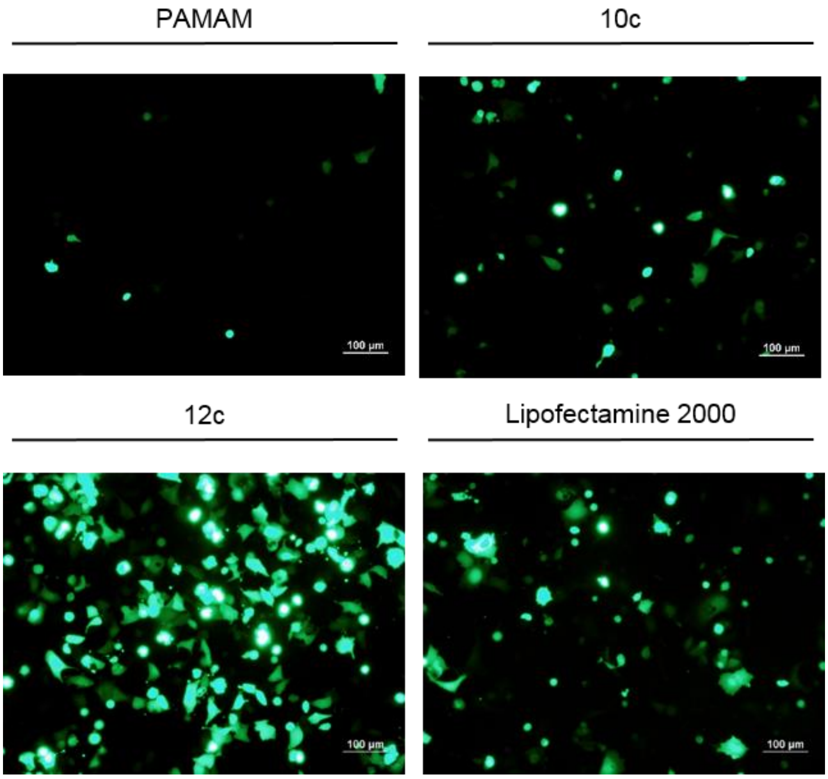

Supplementary Figure 22: EGFP expression representing successful transfection in MCF-7 cell line at optimum N/P ratios respectively. Large EGFP expressing plasmid (10.3 kb) was used for the transfection. Experiment was completed with technical triplicates and biological duplicates.

2.8 Cotransfection

2.8.1 Method of codelivery

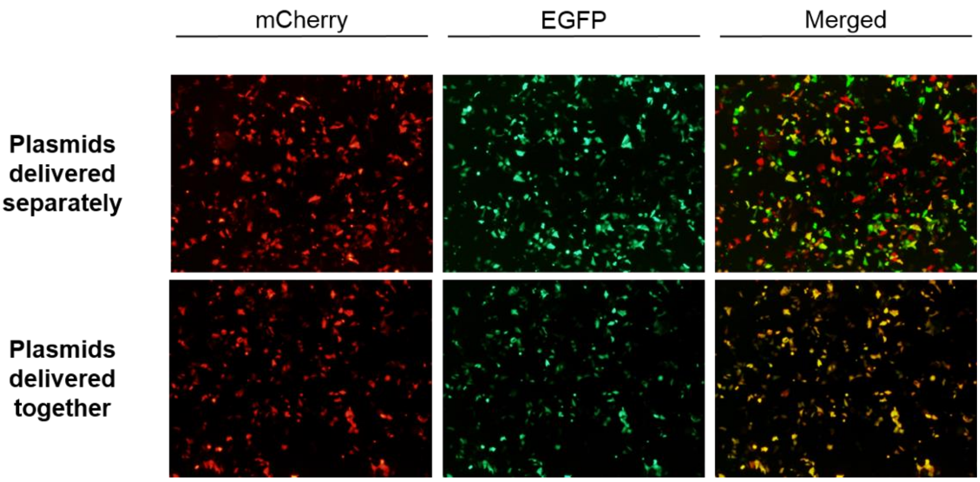

Supplementary Figure 23: Cotransfection of mCherry and EGFP plasmids when delivered separately or delivered together. MCF-7 cells were cotransfected with mCherry and EGFP (both 5.3 kb). Plasmids were either complexed separately before delivery, or mixed and complexed together. Cotransfection efficiency of both plasmids was higher when the plasmids were mixed and complexed together.

2.8.2 Cotransfection using plasmids of equal size

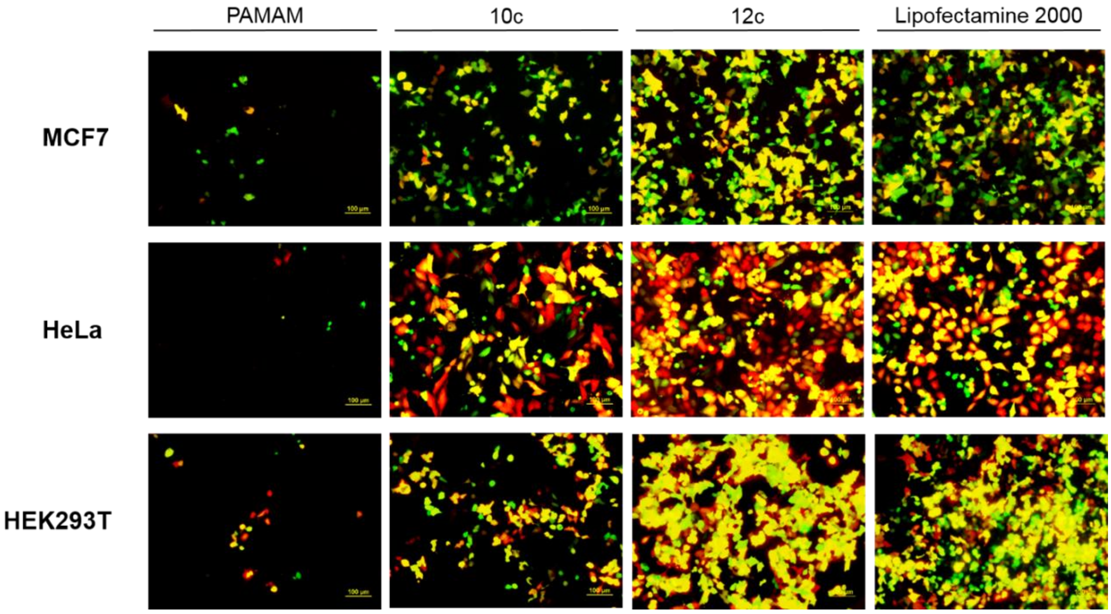

Supplementary Figure 24: Cell lines cotransfected with mCherry and EGFP plasmids (5.3 kb each). Successful delivery of both plasmids results in the cell appearing yellow. Experiment was completed with technical triplicates in each of the cell lines.

2.8.3 Cotransfection using plasmids of different size

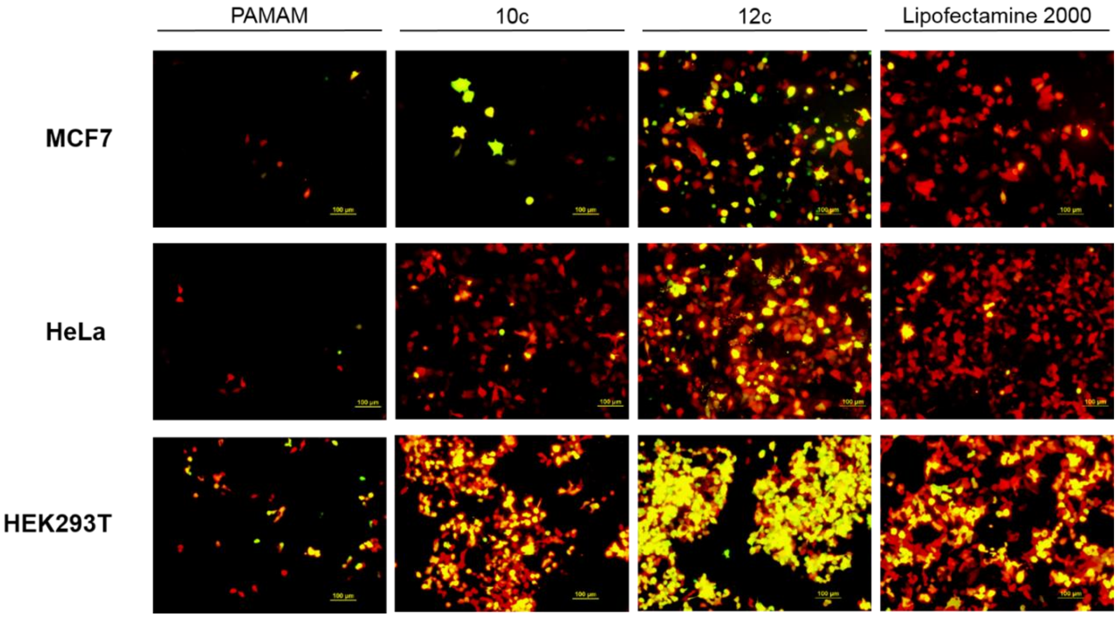

Supplementary Figure 25: Cell lines co-transfected with mCherry (5.3 kb) and EGFP plasmids (10.3 kb). Successful delivery of both plasmids results in the cell appearing yellow. Experiment was completed with technical triplicates in each of the cell lines.

2.8.4 Cotransfection flow cytometry gatings

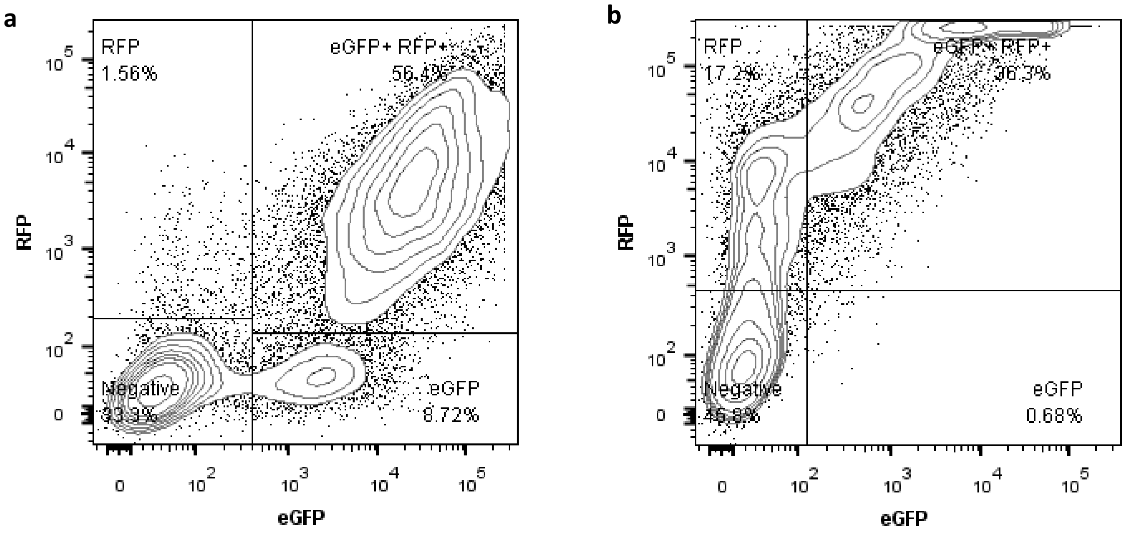

Supplementary Figure 26: Representative flow cytometry gatings for **a**, small plasmid (5.3 kb) co-transfection, and **b**, mixed plasmid (5.3 kb and 10.3 kb) co-transfection experiments in MCF-7 cell line.

2.8.5 Cytotoxicity in cotransfected cell lines

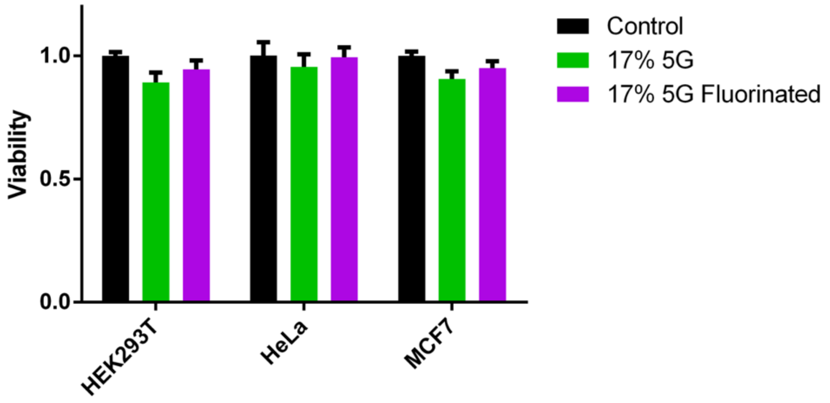

Supplementary Figure 27: Cell viability across multiple cell lines when transfected using **10c** and **12c** polymer formulations. Cells were transfected with 5.3 kb EGFP plasmid at various N/P ratios and cell viability was assessed at 48 h. Culture conditions and polymer concentrations were identical to those used in the transfection experiments. Cell viability did not drop below 90% of the control across all conditions.

2.9 *MASPIN* mRNA quantification

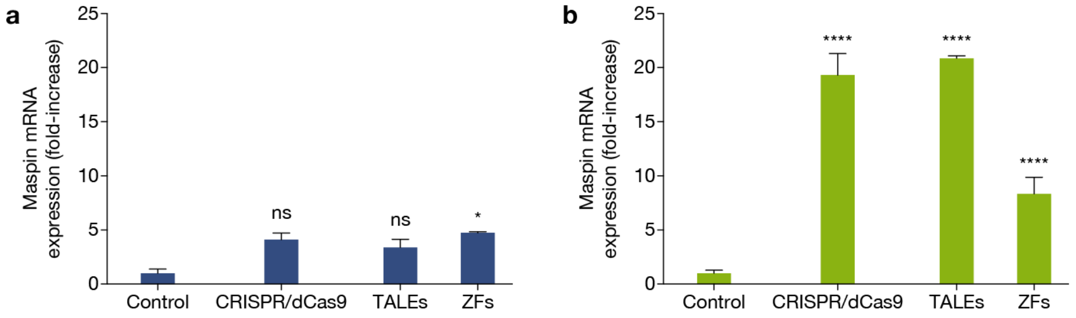

Supplementary Figure 28: Increase in *MASPIN* mRNA expression following delivery of CRISPR/dCas9, TALEs and ZF systems using **a**, Lipofectamine 2000 and **b**, polymer **12c**. Statistical significance was determined in comparison to control, with * $p < 0.05$, ** $p < 0.01$, *** $p < 0.001$, **** $p < 0.0001$, ns $p > 0.05$.

2.10 *MASPIN* protein quantification

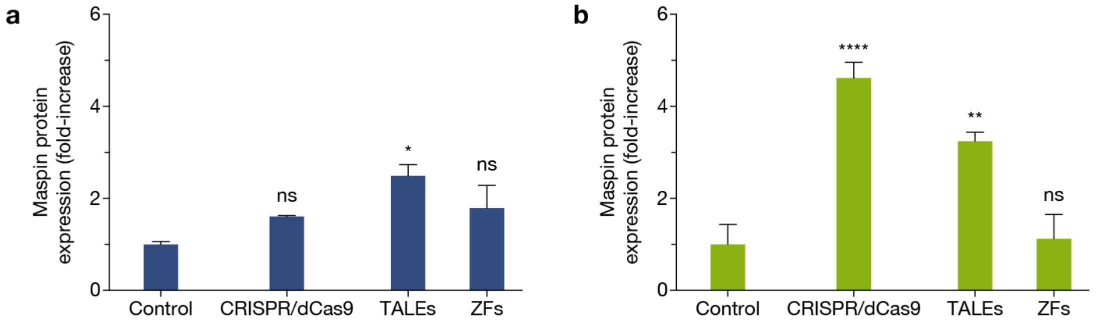

Supplementary Figure 29: Increase in MASPIN protein expression following delivery of CRISPR/dCas9, TALEs and ZF systems using **a**, Lipofectamine 2000 and **b**, polymer **12c**. Statistical significance was determined in comparison to control, with * $p < 0.05$, ** $p < 0.01$, *** $p < 0.001$, **** $p < 0.0001$, ns $p > 0.05$.

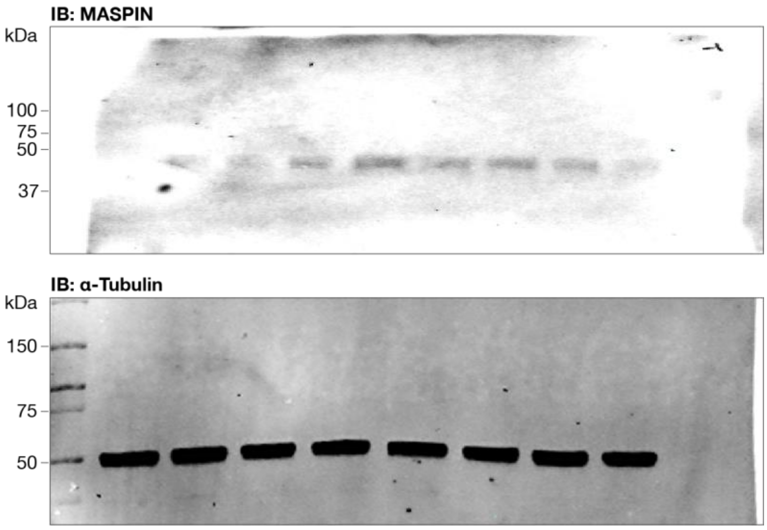

Supplementary Figure 30: Full immunoblots for quantification of MASPIN and α-Tubulin. SDS-PAGE was performed on 10% acrylamide gels and transferred to PVDF membrane.

2.11 Plasmid maps

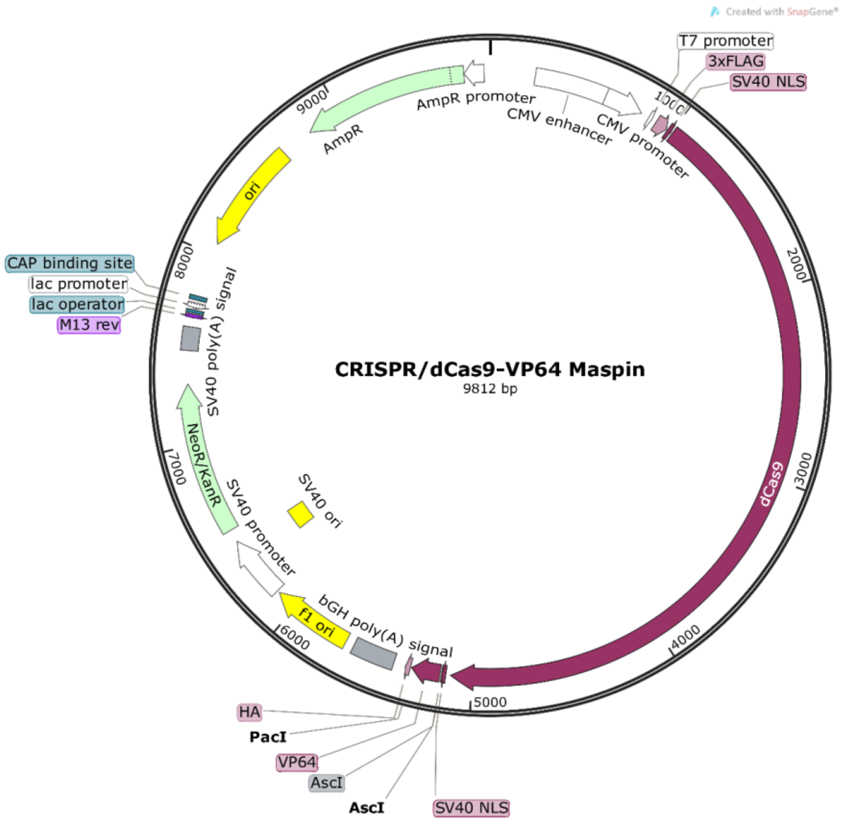

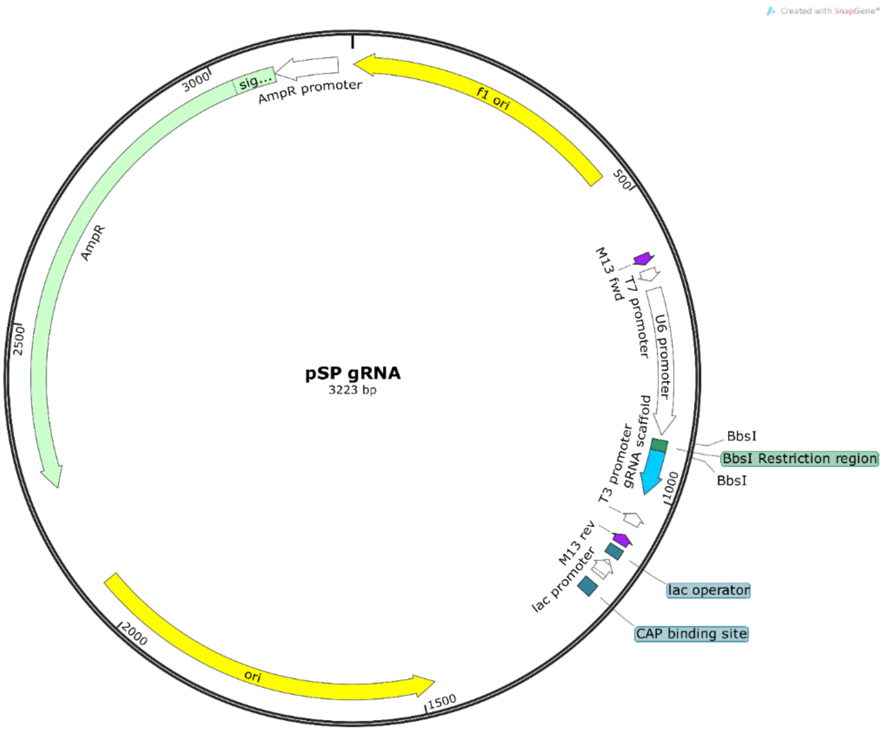

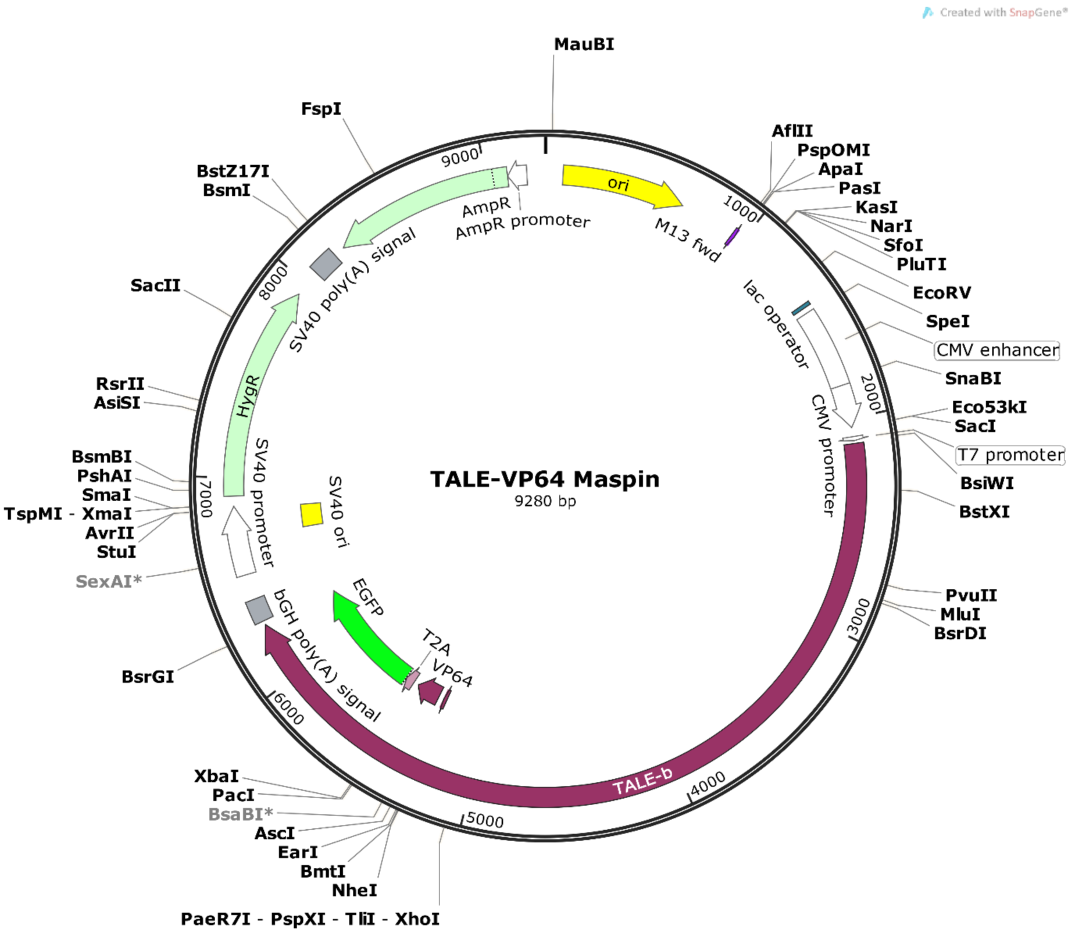
Created with SnapGene®
TALE-VP64 Maspin
9280 bp
MauBI
FspI
BstZ17I
BsmI
SacII
RsrII
AsiSI
BsmBI
PshAI
SmaI
TspMI - XmaI
AvrII
StuI
SexAI*
BsrGI
XbaI
PacI
BsaBI*
AscI
EarI
BmtI
NheI
PaeR7I - PspXI - TliI - XhoI
AflII
PspOMI
ApaI
PasI
KasI
NarI
SfoI
PluTI
EcoRV
SpeI
CMV enhancer
SnaBI
Eco53kI
SacI
T7 promoter
BsiWI
BstXI
PvuII
MluI
BsrDI
ori
M13 fwd
lac operator
CMV promoter
TALE-b
AmpR
AmpR promoter
SV40 poly(A) signal
HygR
SV40 promoter
SV40 ori
bGH poly(A) signal
EGFP
T2A
VP64
1000
2000
3000
4000
5000
6000
7000
8000
9000

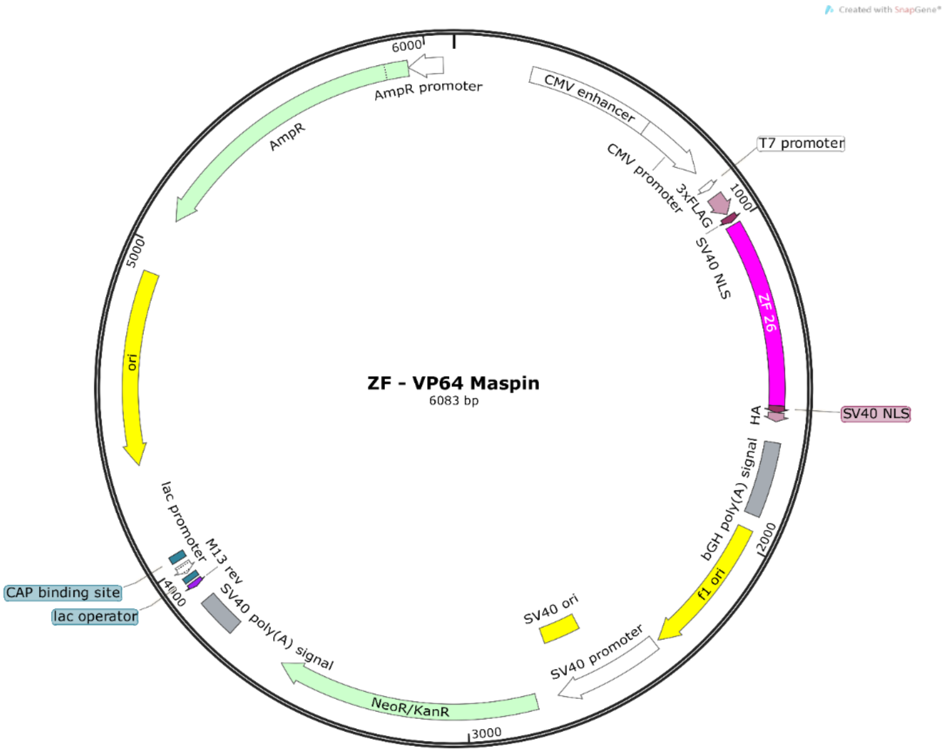
Created with SnapGene®
ZF - VP64 Maspin
6083 bp
AmpR promoter
AmpR
CMV enhancer
CMV promoter
T7 promoter
3xFLAG
SV40 NLS
ZF 26
HA
SV40 NLS
bGH poly(A) signal
f1 ori
SV40 ori
SV40 promoter
NeoR/KanR
SV40 poly(A) signal
M13 rev
lac promoter
CAP binding site
lac operator
ori
1000
2000
3000
4000
5000
6000

3 Summary of Prepared Compounds

Supplementary Table 12: Summary of prepared compounds and their associated shorthand label.

	**Dendron Generation**			
	**Unfluorinated**		**Fluorinated**	
**x**	**4**	**5**	**4**	**5**
**3**	9a	10a	11a	12a
**8**	9b	10b	11b	12b
**17**	9c	10c	11c	12c
**28**	9d	10d	11d	12d
